# 3D printed devices and infrastructure for liquid sample delivery at the European XFEL

**DOI:** 10.1107/S1600577521013370

**Published:** 2022-02-15

**Authors:** Mohammad Vakili, Johan Bielecki, Juraj Knoška, Florian Otte, Huijong Han, Marco Kloos, Robin Schubert, Elisa Delmas, Grant Mills, Raphael de Wijn, Romain Letrun, Simon Dold, Richard Bean, Adam Round, Yoonhee Kim, Frederico A. Lima, Katerina Dörner, Joana Valerio, Michael Heymann, Adrian P. Mancuso, Joachim Schulz

**Affiliations:** a European XFEL, Holzkoppel 4, 22869 Schenefeld, Germany; b Center for Free-Electron Laser Science (CFEL), Deutsches Elektronen-Synchrotron (DESY), Notkestrasse 85, 22607 Hamburg, Germany; c Department of Physics, TU Dortmund, Otto-Hahn-Straße 4, 44221 Dortmund, Germany; d School of Chemical and Physical Sciences, Keele University, Staffordshire ST5 5AZ, United Kingdom; e Institute for Biomaterials and Biomolecular Systems (IBBS), University of Stuttgart, Pfaffenwaldring 57, 70569 Stuttgart, Germany; f Department of Chemistry and Physics, La Trobe Institute for Molecular Science (LIMS), La Trobe University, Melbourne 3086, Australia

**Keywords:** FEL physics, instrumentation, microfluidics, liquid jets, high-viscosity extrusion, aerosols, rapid mixing, sample delivery, X-ray scattering, crystallography, single-particle imaging

## Abstract

The Sample Environment and Characterization (SEC) group of the European X-ray Free-Electron Laser (EuXFEL) develops sample delivery systems for the various scientific instruments, including systems for the injection of liquid samples that enable serial femtosecond X-ray crystallography (SFX) and single-particle imaging (SPI) experiments, among others. For rapid prototyping of various device types and materials, sub-micrometre precision 3D printers are used to address the specific experimental conditions of SFX and SPI by providing a large number of devices with reliable performance. This work presents the current pool of 3D printed liquid sample delivery devices, based on the two-photon polymerization (2PP) technique. These devices encompass gas dynamic virtual nozzles (GDVNs), mixing-GDVNs, high-viscosity extruders (HVEs) and electrospray conical capillary tips (CCTs) with highly reproducible geometric features that are suitable for time-resolved SFX and SPI experiments at XFEL facilities. Liquid sample injection setups and infrastructure on the Single Particles, Clusters, and Biomolecules and Serial Femtosecond Crystallography (SPB/SFX) instrument are described, this being the instrument which is designated for biological structure determination at the EuXFEL.

## Abstract 2

Presented here are 3D printed sample delivery devices for precise control over fluids and the generation of micrometre-sized gas-focused liquid jets, high-viscosity streams and near-monodisperse droplets suitable for X-ray scattering experiments on X-ray free-electron laser (XFEL) instruments.

## 1. Introduction

### 1.1. Motivation

X-ray free-electron lasers (XFELs) provide short X-ray pulses with extremely high peak brilliance and spatial beam coherence for advanced X-ray imaging and spectroscopy experiments (Decking *et al.*, 2020 ▸; Kern *et al.*, 2013 ▸; Levantino *et al.*, 2015 ▸; Katayama *et al.*, 2019 ▸). In a single pulse, typically a few tens of femtoseconds long, an XFEL can provide the order of magnitude of photons that a synchrotron delivers in a second. This places stringent requirements on the XFEL sample delivery that the Sample Environment and Characterization (SEC) group of the European X-ray Free-Electron Laser (EuXFEL) provides to its users.

Since the EuXFEL started user operation in 2017, the SEC group supports users in sample preparation, characterization and delivery, and is dedicated to continuous refinement and improvement of these procedures for all scientific instruments. This article will introduce the available experimental conditions and infrastructure pertaining to X-ray and sample delivery, in particular with a focus on:

(i) Describing the EuXFEL facility specifications and sample injection environments/requirements on the SPB/SFX instrument.

(ii) Summarizing the SEC’s current pool of high-throughput 2PP-3D printed devices tailored for liquid sample injection, with details of design parameters, fabrication and performance.

(iii) Discussion of novel experimental data that are of importance for planning XFEL experiments (obtainable liquid jet velocities and microdroplet diameters, diffraction of biological samples, and material properties of the utilized photoresists for device fabrication).

One imaging method in particular, serial femtosecond X-ray crystallography (SFX), benefits from the short pulses and high photon flux of an XFEL to outrun the radiation damage of crystals that would limit the resolution in conventional technologies (Schlichting, 2015 ▸). This diffraction-before-destruction approach makes use of the fact that radiation damage takes time to manifest after X-ray excitation. The scattered photons impart structural information before the crystal disintegrates completely. Furthermore, the high intensity allows the study of crystals that are too small for conventional macromolecular crystallography (MX) beamlines. Compared with MX, where the crystals are usually under cryogenic conditions, SFX analyses crystals under near-native conditions at room temperature. This eliminates possible structural changes due to freezing. Ultimately, this method may be used for structure determination without the need to crystalize at all (Neutze *et al.*, 2000 ▸; Seibert *et al.*, 2011 ▸; Bielecki *et al.*, 2020 ▸).

In combination with rapid mixing and/or femtosecond laser excitation, XFELs can resolve intermediate states in chemical reactions (Brändén & Neutze, 2021 ▸). The combination of multiple methods, such as resonant and non-resonant X-ray absorption, scattering, and fluorescence at different delay times between the exciting optical pulse and probing X-ray pulse, provides the data for assembling molecular movies that show small molecules interacting with proteins (Kern *et al.*, 2013 ▸; Canton *et al.*, 2015 ▸; Pandey, Bean *et al.*, 2020 ▸; Pandey, Poudyal *et al.*, 2020 ▸; Nass *et al.*, 2020 ▸).

XFELs provide several tens to several thousands of pulses per second and therefore reduce the necessary sampling time for a full data set considerably. The high peak power of XFELs, however, potentially destroys the sample in a single shot. Therefore, the facilitation of devices that ensure continuous sample replenishment, compatible with these high repetition rates, is of utmost importance (Zhao *et al.*, 2019 ▸; Cheng, 2020 ▸).

Today, 3D printing based on two-photon polymerization (2PP) enables rapid, reproducible and high-throughput fabrication of the required sample delivery devices (Nelson *et al.*, 2016 ▸). Moreover, it allows nearly unlimited design flexibility with a convenient implementation of geometric variations (diameter of liquid/gas apertures, spacing between apertures, multi-orifice flow-focusing and mixing configurations, droplet generation *etc.*), while maintaining a low mater­ial footprint (Echelmeier, Kim *et al.*, 2019 ▸; Knoška *et al.*, 2020 ▸; Nazari *et al.*, 2020 ▸; Šarler *et al.*, 2021 ▸).

The increasing availability of 2PP-3D printers at research facilities enables new avenues for collaboration. Most importantly, digital designs can be easily exchanged. With this work, we aim to contribute to the growing micro/nanofabrication community by providing our 3D device design files for download through a GitHub repository, accessible at https://github.com/flmiot/EuXFEL-designs. We hope to make a positive impact on the quality of sample delivery devices, not only by fuelling new design ideas and improvements but also by allowing users to facilitate sample injection tests at their home institute prior to the beamline experiment.

### 1.2. The SPB/SFX instrument

The Single Particles, Clusters, and Biomolecules and Serial Femtosecond Crystallography (SPB/SFX) instrument is one of the hard X-ray scientific endstations after the SASE1 undulator, and is dedicated to SFX and single-particle imaging (SPI) of biomacromolecules and complexes such as viruses, organelles and cells (Mancuso *et al.*, 2019 ▸). Two sets of mirrors are available on the SPB/SFX instrument, either micrometre- or nanometre-scale Kirkpatrick–Baez focusing mirrors, depending on the required X-ray focus size (Bean *et al.*, 2016 ▸).

Two different X-ray interaction regions are accessible to users. First, the Interaction Region Upstream (IRU) in high vacuum, suitable for up to 4.5 MHz X-ray repetition rates, with a 1M adaptive gain integrating pixel detector (AGIPD), allows the collection of up to 3520 frames per second with a dynamic range specification of 10^4^ photons per pulse per pixel at 12 keV (Allahgholi *et al.*, 2019 ▸). Possible sample-to-AGIPD distances are 12–550 cm. Second, the Interaction Region Downstream (IRD) (Round *et al.*, 2022, in preparation) uses refocused X-rays enabled by a set of compound refractive lenses (CRLs), currently in air or inside a helium-purged sample chamber presently limited to 10 Hz operation, where the detector is a 4M JUNGFRAU (Mozzanica *et al.*, 2018 ▸).

Various liquid sample delivery methods are supported on the SPB/SFX instrument, including those described in this paper, which are employed predominantly at the IRU (Schulz *et al.*, 2019 ▸). The IRD is mostly devoted to high-viscosity extrusion experiments at atmospheric pressure, as well as fixed-target samples for crystallography. Future developments will also allow custom user setups.

SFX, in general, offers great opportunities in structural biology, as it allows the determination of protein structures at the atomic scale that can facilitate a structure-based drug design (Mishin *et al.*, 2019 ▸). However, beam time at XFELs is very limited compared with what is available on synchrotron beamlines. For this reason, SFX experiments on SPB/SFX in particular must use the XFEL’s characteristics to study macromolecular crystals that are too small for synchrotron analysis (crystal sizes in the micrometre to sub-micrometre range) or that are very sensitive to radiation damage (*e.g.* metalloproteins), or for time-resolved investigations as a means of understanding a systems’ dynamics, triggered either by diffusive mixing (down to a few milliseconds) or laser-driven pump–probe schemes (microseconds and shorter timescales) (Orville, 2020 ▸; Mills *et al.*, 2020 ▸).

For such laser applications, several optical sources are available. The capabilities, which are updated on the instrument’s website before each proposal round, range from commercial nanosecond Nd:YAG lasers and optical parametric oscillators (OPO) operating at 10 Hz with a tuneable wavelength range (210–2400 nm) to the EuXFEL femto­second pump–probe laser operating up to megahertz repetition rates (Palmer *et al.*, 2019 ▸). The latter can be coupled to an optical parametric amplifier (OPA) to provide an extended tunability range from 250 nm to 15 µm.

### 1.3. X-ray pulse patterns and sample delivery constraints

**Figure 1:**
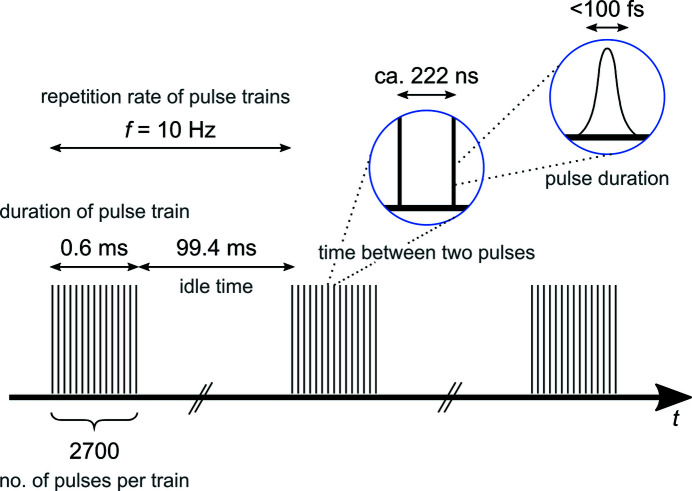
The high repetition rate X-ray pulse pattern at the EuXFEL. X-ray pulses arrive in 10 Hz trains at the sample and each train can provide up to 2700 ultrashort pulses.

The EuXFEL provides X-ray radiation with a unique pulse pattern (Fig. 1 ▸). It delivers up to 2700 femtosecond-long pulses in 600 µs long trains with a train repetition rate of 10 Hz, shared between the active scientific instruments. Between two adjacent pulses within a train, as little as *ca* 222 ns elapses, which corresponds to a maximum 4.5 MHz intra-train repetition rate of the X-ray pulses. The accelerator is equipped with a magnetic kicker system that can deflect single electron bunches into a dump, allowing for a fully flexible choice of the pulse pattern in each train. Typically, a 1.13 MHz (890 ns, 352 pulses) operation is chosen on SPB/SFX.

Optical lasers can be used to pump the injected sample (*e.g.* for photoactivation) prior to being probed by the X-ray laser. Therefore, the liquid jet has to ensure that each sample volume of interest is replenished rapidly after each probe pulse (or between each pump–probe cycle). In general, the X-ray beam size (*ca* 3–6 µm on SPB/SFX) is much smaller than the optical laser beam size (*ca* 30–1000 µm). Hence, the sample volume needs to be displaced by more than the optical laser beam size (pump pulse) before the next pump or probe pulse arrives.

Another important constraint is the enormous energy deposited in a single ultrashort X-ray pulse (*ca* 2.5 mJ at SASE1), which vaporizes a segment of the jet, leading to gaps in the liquid column. Each explosion further generates a shock wave that propagates both upstream and downstream (Stan *et al.*, 2016 ▸; Grünbein *et al.*, 2021 ▸). The spacing between two gaps is usually of the order of several tens of micrometres (Chavas *et al.*, 2015 ▸) and roughly describes the displacement between two intra-train pulses, which allows the determination of jet velocities (Grünbein, Bielecki *et al.*, 2018 ▸; Wiedorn, Oberthür *et al.*, 2018 ▸). The distance between two gaps (and consequently the jet velocity) decreases with increasing liquid flow rates, while it increases for larger gas flow rates.

At any point along a liquid jet, the jet velocity is given by the volumetric flow rate divided by the jet cross-sectional area. For a chosen liquid flow rate, the jet velocity therefore varies as the inverse square of the jet diameter. The free jet diameter is generally observed to increase with increasing flow rate, and this must be considered when scaling jet velocities to different flow rates. Conversely, fast jets, which are required for FEL experiments at high repetition rates, are observed at low liquid flow rates (≤20 µl min^−1^) and high helium mass flow rates (≥20 mg min^−1^) which accelerate the liquid.

Since the EuXFEL accelerator can run at different repetition rates, the minimal required jet velocity depends not only on the pump and probe beam sizes but also on the utilized machine settings. The required jet velocity can therefore be regarded as the product of the displacement between two pulses, Δ*x*, and the intra-train repetition rate *f*_pulse_. Table 1 ▸ provides a list of EuXFEL instrument configurations with four different pulse patterns, the resulting beam parameters and the required jet velocities for efficient sample replenishment.

For instance, as shown in Table 1 ▸, a 44 µm spacing was observed in a 5 µm wide liquid jet generated with a 75–60–75 GDVN [*Q*_water_ = 30 µl min^−1^, *p*_He_ = 450 psi (34 mg min^−1^); 1 psi ≃ 6893 Pa] during 1.13 MHz operation (with 300 pulses per train). When reducing *f*_pulse_ by half (0.564 MHz), the Δ*x* of the same liquid jet doubled, as expected. Lower repetition rates allow for probing slower (wider) jets. In general, jets with velocities of 25 m s^−1^ and greater are fast enough for 564 kHz operation, while 1.13 MHz operation should be considered only when providing jets with *v* ≥ 45 m s^−1^. On account of this relation between the velocities and pulse repetition rates, it is theorized that 4.51 MHz operation requires jets with *v* ≃ 150 m s^−1^. However, it is worth noting that thinner jets produce smaller gaps and, consequently, would manage a higher repetition rate than one would expect from comparing them with data from a thicker jet (Stan *et al.*, 2016 ▸; Wiedorn, Awel *et al.*, 2018 ▸).

The required jet velocities for megahertz data collection can be quite discouraging, as high pressures for the focusing He are needed to provide such thin and fast jets. The He flow rate, on the other hand, directly impacts the vacuum level in the sample chamber, which must remain below 2 × 10^−4^ mbar (1 bar = 100 000 Pa) to allow AGIPD operation (see the supporting information, Section S3.4). Moreover, the pump lasers regularly run at an integer divisor of the maximum X-ray repetition rate (*e.g.* only every second or third X-ray pulse is ‘pumped’). Assuming that the X-ray pulse creates negligible distortion of the liquid jet (or that distortion is acceptable), the required jet velocity can be much lower. This can be estimated by setting *f*_pulse_ to a value that is equal to the pump laser repetition rate.

### 1.4. Sample delivery systems

#### 1.4.1. GDVNs and DFFNs

Freely flowing liquid columns (liquid jets) achieve the required sample replenishment for SFX, while allowing high signal-to-noise ratios due to the low scattering background of water. Apart from its well established application for SFX (Echelmeier, Sonker *et al.*, 2019 ▸), liquid jet-based sample delivery is today also used in synchrotron-based X-ray scattering experiments such as small-angle X-ray scattering (SAXS) (Marmiroli *et al.*, 2009 ▸; Saldanha *et al.*, 2017 ▸), wide-angle X-ray scattering (WAXS) (Spence *et al.*, 2012 ▸; Steinke *et al.*, 2016 ▸) and serial synchrotron crystallography (SSX) (Stellato *et al.*, 2014 ▸; Pearson & Mehrabi, 2020 ▸).

The increased use of liquid jet devices was enabled by the development of the gas dynamic virtual nozzle (GDVN) (DePonte *et al.*, 2008 ▸). Here, the co-flow of a sheathing gas constitutes a ‘virtual’ nozzle for the liquid to flow through, which leads to the acceleration and convergence of the liquid. This converging fluid meniscus results in a cylindrical jet with diameters down to a few micrometres. The reduced jet diameter facilitates stable sample injection at very low liquid flow rates of the order of a few tens of microlitres per minute. GDVNs have paved the way for SFX experiments at FELs (Chapman *et al.*, 2011 ▸; Boutet *et al.*, 2012 ▸; Gisriel *et al.*, 2019 ▸). However, extensive manual fabrication steps render production scale up difficult and often yield poor device reproducibility for such glass-based devices. While many other fabrication methods have been reported since the advent of glass-based nozzles (Hejazian *et al.*, 2021 ▸), the introduction of stereolithography-based 3D printing of high-resolution nozzles from photocured acrylate-based polymers helped streamline the fabrication process (Nelson *et al.*, 2016 ▸). This material- and time-saving manner has recently led to highly compact nozzles and micromixers suitable for SFX (Knoška *et al.*, 2020 ▸). Extending these developments, we now report new nozzle geometries and device types to cater for a wider range of samples and probing schemes.

In general, GDVNs require sample flow rates up to 20 µl min^−1^ to produce a stable jet of protein crystal suspensions at concentrations in excess of 10^11^ crystals per millilitre. However, many biological samples cannot be produced in such large quantities. With their use of a third orifice for a focusing sheath liquid, double-flow focusing nozzles (DFFNs) address the strong need to reduce sample consumption, while improving the nozzles’ operational stability (Oberthuer *et al.*, 2017 ▸). In the DFFN, an inner (sample) stream is focused by a coaxial faster-flowing outer liquid (usually ethanol) and propagates as a thin jet within the ethanol jet. The ethanol jet itself is then focused by coaxial gas flow (as in the traditional GDVN). As the jet is primarily formed by the sheath liquid, the sample consumption can be reduced compared with a regular GDVN.

#### 1.4.2. Micromixers

Microfluidic environments allow precise control over fluids due to the inherent laminar flow (low Reynold numbers in micrometre-sized channels) (Whitesides, 2006 ▸). The short pathways in microchannels promote fast and controlled diffusion-based mixing, which is essential for a systematic analysis of mixing-induced reaction kinetics and structural transitions of (bio)macromolecules (Köster & Pfohl, 2012 ▸; Ghazal *et al.*, 2016 ▸; Sui & Perry, 2017 ▸).

The temporal structural evolution during mixing, as set by the microchannel geometry and applied continuous-flow regime, can be mapped onto different downstream positions for time-resolved studies (With *et al.*, 2014 ▸) and, in combination with microfocused X-rays, timescales down to the sub-millisecond scale can be accessed (Park *et al.*, 2006 ▸, 2008 ▸). In this manner, the conjunction of microchannels and GDVN-based injection (Wang *et al.*, 2014 ▸; Trebbin *et al.*, 2014 ▸) can enable mix-and-inject SFX. Here, for instance, rapid mixing of enzyme crystals with their substrates is achieved immediately prior to jet-based sample injection (Schmidt, 2013 ▸, 2020 ▸; Pandey *et al.*, 2021 ▸).

To accommodate such experiments routinely, we have adopted the glass-based microfluidic mixing injector reported by Calvey *et al.* (2016 ▸) and translated it into a single-piece 2PP-3D printed device. Our micromixer–GDVN hybrid provides a 100 µm wide circular channel, small enough to ensure low sample consumption and fast diffusion but large enough to allow retention for the majority of obtainable crystal sizes, leading into a 75–60–75 GDVN (type B; see Table 2 ▸). For the channel length, variations of 2.12 mm and 332 µm, respectively, are currently available.

The mixing of two fluids (*e.g.* sample with reactant) is enabled by two liquid channels that converge and intersect via concentric cones, one having a 75 µm wide aperture and the other (the surrounding reactant) a 180 µm wide opening which tapers down to a 100 µm wide constriction. The promoted 3D hydro­dynamic flow-focusing shortens the diffusion pathway for the sheathing reactant molecules into the sample stream (Knight *et al.*, 1998 ▸). The design further minimizes the Taylor dispersion, as it positions the sample in the centre of the 3D parabolic flow profile to ensure minimal velocity mismatch between fluid layers and avoid inhomogeneous mixing (Vakili *et al.*, 2019 ▸). Moreover, the sample stringently avoids wall contact which could result in wall agglomerations and consequent channel clogging.

**Figure 2:**
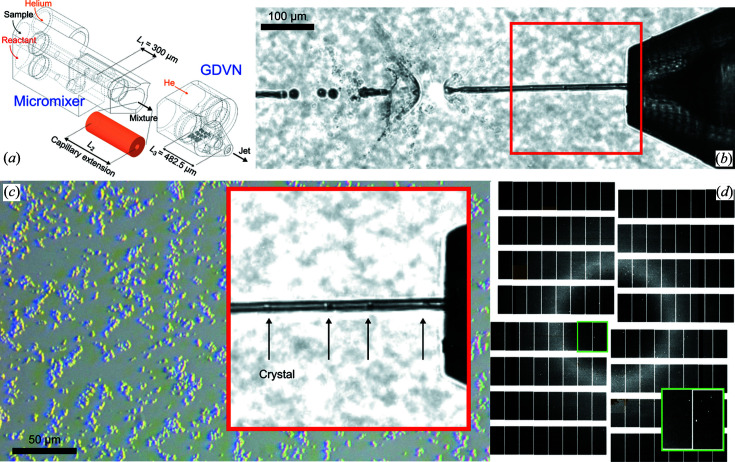
(*a*) An overview of the 2PP-3D printed mix-and-inject device (*i.e.* mixing-GDVN) consisting of a micromixer, a GDVN (type C) and a connective capillary in between. The capillary extension was chosen to be *L*_2_ = 28 mm. (*b*) A microscopy image showing lysozyme crystal delivery on the SPB/SFX instrument (10× magnification, NA 0.28, pixel size ∼0.65 µm). Upstream from the depicted X-ray interaction region, the crystals enter from the main channel of the mixing device at 7 µl min^−1^ and are flow-focused by pure water entering from the side channel at 70 µl min^−1^. Downstream, the 11-fold diluted sample then enters the GDVN (type C, 100 µm liquid orifice). With a helium pressure of 550 psi (*Q*_g_ = 34 mg min^−1^), a liquid jet of 7.5 µm in diameter delivers the crystals into the X-ray focus. With our prediction formula [Fig. 4(*b*)], the jet velocity was determined to be 30.5 m s^−1^. (*c*) A microscopy image of the lysozyme crystal dispersion from the utilized sample reservoir showing the near-monodisperse microcrystals. (*d*) A background-corrected detector image of lysozyme diffraction from the same 11-fold dilution collected on the AGIPD 1M detector. The detector (pixel size is 200 µm × 200 µm) consists of four movable quadrants, each quadrant consisting of four static independent modules. Each module is 26 mm × 103 mm (128 × 512 pixels) large and consists of 2 × 8 ASICs (application-specific integrated circuits). The magnified region (green rectangle) shows Bragg reflections within 2 × 2 ASICs.

Throughout the microchannel, highly efficient and homogeneous mixing then occurs via 3D diffusion across the flow direction, and retention times of several tens to several hundreds of milliseconds can be controlled. To access even longer retention/mixing times, a modular assembly approach can be pursued in which a printed micromixer and GDVN are connected by a liquid capillary extension of custom length [Fig. 2 ▸(*a*)]. For these micromixers, a second design variation provides a diameter ratio of 100:231.7 µm leading into a 200 µm wide mixing channel for larger crystals and/or more viscous buffers.

More complex channel architectures, such as the Kenics mixer (type JKMH_10), invoke a series of helical elements for repeated flow splitting/stretching to increase the diffusive interface exponentially with each additional element. Mixing time point uniformity/dispersion becomes thus more dependent on the spatial location along the mixer and highly insensitive to flow rate fluctuations, as comparable mixing can be observed at the same channel section for liquid flow rates and viscosities diverging by as much as an order of magnitude (Knoška *et al.*, 2020 ▸).

#### 1.4.3. High-viscosity extruders

An important sample class for SFX-based structure determination are membrane proteins involved in ion transport, cell adhesion and signal transduction (Standfuss, 2019 ▸). However, the hydrophobicity of these proteins makes it difficult to produce crystals in aqueous media as these highly vulnerable proteins would need to be removed from their native lipidic environment, thus exposing them to conditions that are prone to denaturation, aggregation and/or degradation. An important viscous medium for growing and delivering such proteins is a membrane-mimetic medium known as the lipidic cubic phase (LCP) (Landau & Rosenbusch, 1996 ▸). Here, lipids, water and proteins form a complex 3D array, which is pervaded by a system of aqueous channels (Li & Caffrey, 2020 ▸).

Because of its gel-like nature and very high viscosity, LCP is incompatible with GDVNs and necessitates a new approach to generate micrometre-sized sample streams. To address this, an injector system composed of a hydraulic cylinder, which is driven by a high-performance liquid chromatography (HPLC) pump, a sample reservoir and a glass-based nozzle tip was developed (Weierstall *et al.*, 2014 ▸). Here, low-pressured coaxial helium flow along the inner capillary in the tip stabilizes the liquid stream rather than compressing it.

In recent years, high-viscosity extrusion (HVE) has thus enabled valuable insights into the structure and dynamics of various membrane proteins embedded in LCP and other mineral oil-based or polymer-based viscous media (Botha *et al.*, 2015 ▸; Nogly *et al.*, 2015 ▸, 2016 ▸; Kovácsová *et al.*, 2017 ▸), whereas the core concepts of Weierstall’s original injector design have remained largely unaltered. However, a method that allows a quick exchange of sample reservoirs without the need to disassemble the entire injection system would be highly desirable. With this rationale, Sugahara *et al.* (2015 ▸) introduced an extrusion of crystals directly from gas-tight and durable glass syringes which can be combined with quartz capillaries or polyimide chips (Nam, 2020 ▸; Nam & Cho, 2021 ▸).

In general, HVE injectors produce very wide streams (roughly equal in diameter to the inner diameter of the sample line) of crystal suspensions with two orders of magnitude lower flow rates than GDVNs. They run with flow rates between 0.05 and 2.0 µl min^−1^ (Nogly *et al.*, 2016 ▸), which result in flow velocities of 0.1–4.2 mm s^−1^ (when considering a 100 µm ID capillary), comparable with tape drive delivery velocities (Beyerlein *et al.*, 2017 ▸).

These sample-conserving stream velocities are suitable for the 10 Hz repetition rate of the X-ray pulse trains at the EuXFEL. However, it is necessary that each pulse train provides only a single pulse, as multiple pulses within the train would hit the same crystal. On account of its compatibility with millisecond X-ray exposures, HVE is frequently applied at synchrotrons (Shilova *et al.*, 2020 ▸).

Today, we employ 2PP-3D printed HVE tips that exactly fit into stainless steel tubing of ID 0.03 inch (1/16 inch OD, IDEX U-115), thus making them compatible with conventional HVE injector systems. The printed extruders provide concentric liquid/gas access ports and inner diameters of 50, 75 or 100 µm for the liquid channel, which extends the 50 mm long access capillary. The reproducible nature of 2PP-3D printing allows for conical tips with an extremely high level of symmetry, which, for instance, prevents curling up of the sample stream due to unevenly distributed gas flow across the flow direction. Our 75 µm ID tips (Fig. S4) can run LCP stably with flow rates as low as 0.3 µl min^−1^ (*v* ≃ 1 mm s^−1^) with a 15 psi helium co-flow (corresponding to a gas flow rate of 11 mg min^−1^ in a 100 µm ID capillary).

#### 1.4.4. Electrosprayers for aerosolization

Single-particle imaging allows for the high-resolution structure determination of isolated molecules, viruses and cells, without the need for preparing crystals, which is often the bottleneck in bio­macro­molecular structure determination (Neutze *et al.*, 2000 ▸; Seibert *et al.*, 2011 ▸). To compensate for the low signal-to-noise ratios of the weakly scattering non-crystalline samples, near background-free sample injection has to be provided (Kirian *et al.*, 2015 ▸).

In this context, current state-of-the-art sample delivery makes use of aerosolized samples that are transported into the X-ray interaction region by an aerodynamic lens (Liu *et al.*, 1995 ▸; Hantke *et al.*, 2018 ▸). The aerosolized sample is produced by nebulizing the sample solution into small droplets which, after evaporation, leave behind gas phase particles. The droplets should be as small as possible to minimize solution residues on the sample particles after evaporation (Bielecki *et al.*, 2019 ▸). Droplets smaller than 100 nm can be produced by the break up of an electrospray (ES) jet. The ES jet is produced at the apex of the electrostatically stable Taylor cone, which in turn is produced when an electric field is applied to the surface of a conductive liquid (Taylor, 1964 ▸).

A prerequisite for effective Taylor cone formation is the external shape of the capillary tip (Chowdhury & Chait, 1991 ▸), which up to now has been realized via highly manual grinding steps. Usually, the capillaries were conically ground at an attack angle of 20–30° until the tip of the capillary had a plateau with sufficient surface area to support a stable Taylor cone (Chen *et al.*, 1995 ▸). These geometric constraints can also be easily introduced by 2PP-3D printed tips with improved reproducibility and greater flexibility in future design enhancements. The conical capillary tips (CCT) presented here provide an access port to receive the 360 µm wide capillary for liquid, a 40 µm inner diameter to match the feeding capillary ID, and a conical exit for effective Taylor cone formation. Capillary grinding, which is prone to manual error, is avoided. Moreover, IDs down to 10 µm can be obtained for providing even smaller droplets which can achieve very gentle aerosolization with reduced solvent contamination after droplet evaporation.

## 2. Experimental

### 2.1. Design parameters

All microfluidic devices were designed to accommodate a connectivity to 360 µm OD fused silica capillaries for the fluid feed. Various orifice sizes allow the generation of wider/smaller jets, which correspond to slower/faster jets. Small-orifice GDVNs are capable of generating extremely thin (∼1 µm) and fast jets that recover in time for the next pulse during megahertz beam operation. The wide jets generated by nozzle types D and E (with jet velocities below 20 m s^−1^) allow, for instance, atmospheric operation at synchrotron sources, *e.g.* for scattering experiments on colloidal systems (Lehmkühler *et al.*, 2017 ▸; Markmann *et al.*, 2020 ▸). Table 2 ▸ provides a comprehensive list of the sample delivery devices currently available at the EuXFEL. Details of the device fabrication (2PP-3D printing and assembly) are listed in the supporting information. Device characterization, assembly and testing proceeded in the immediate vicinity of the beamlines in the XBI (XFEL Biology Infrastructure) laboratories (Han *et al.*, 2021 ▸).

## 3. Results and discussion

### 3.1. Liquid jet velocities

The velocities of the liquid jets generated by 2PP-3D printed GDVNs were determined by two experimental methods in high vacuum. In the first method (‘droplet PIV’), the droplets formed after jet break up are exposed twice in the same camera image by two consecutive nanosecond laser pulses separated in time by a delay Δ*t* (Grünbein, Shoeman *et al.*, 2018 ▸). In the resulting image, each droplet will be imaged twice with a spatial separation Δ*x*, and the droplet velocity is given by *v* = Δ*x*/Δ*t*. The time delay was adjusted by a delay generator and verified by the signal from a photodiode mounted inside the vacuum chamber.

**Figure 3:**
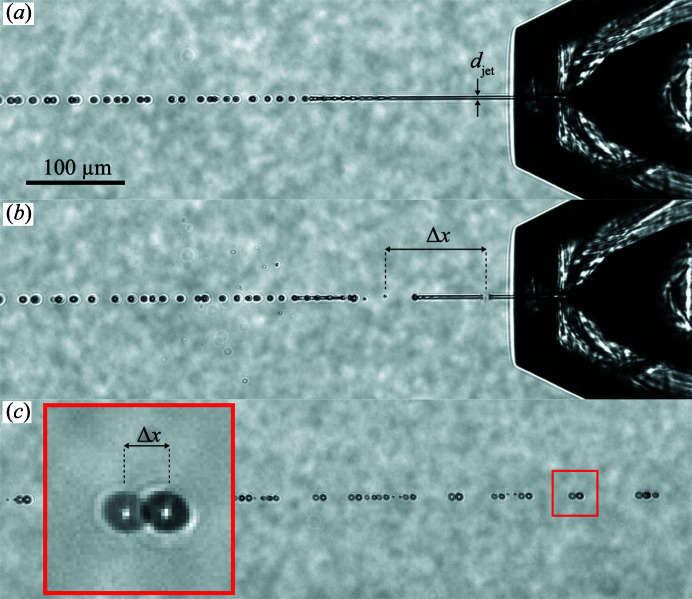
Side view images (10× magnification, NA 0.28, 1075 × 310 pixels^2^ detection area, pixel size ∼0.65 µm) taken inside the SPB/SFX sample chamber, each depicting a thin liquid jet (water) generated by a 75–60–75 (µm) GDVN using a liquid flow rate of *Q*_l_ = 10 µl min^−1^ and an applied gas pressure of *p*_He_ = 400 psi (*Q*_g_ = 25 mg min^−1^). (*a*) The liquid jet in the absence of X-rays. (*b*) X-ray pulses arrive at *f* = 0.564 MHz (with 30 pulses per train) and create gaps in the liquid column (gap-to-gap spacing Δ*x* = 104.7 µm). The X-ray interaction with the jet hence reveals a jet velocity of *v*_jet_ = Δ*x* × *f*_pulse_ = 59.1 m s^−1^. (*c*) Two optical lasers (*λ* = 532 nm), each with a 5 ns pulse duration, illuminate the droplets 2 mm downstream of the jet region. The delay time between the two laser pulses is 119 ns and reveals a droplet displacement of Δ*x* = 7.2 µm. Therefore, dual-pulse laser illumination reveals a droplet velocity of *v*_droplet_ = Δ*x*/Δ*t*_opt.pulse_ = 60.1 m s^−1^. The determined jet velocities imply a jet diameter of *d*_jet_ = [4*Q*_l_/(π*v*)]^1/2^ ≃ 2 µm.

The second method (‘jet explosion’) instead uses double exposure by two consecutive X-ray pulses and a single optical laser pulse. Each X-ray pulse induces an explosion of the jet, causing shock waves that travel through the liquid column (Wiedorn, Oberthür *et al.*, 2018 ▸; Blaj *et al.*, 2019 ▸). In the inertial frame of the liquid jet, the two shock waves travel symmetrically upstream and downstream of the flow direction and leave behind a gap without liquid. Consequently, the midpoint of this gap continues to travel at the velocity of the liquid jet. Thus, by capturing two consecutive jet explosions in the same camera image, the spatial and temporal separation of the two midpoints can be used to determine the jet velocity, in this case *v* = Δ*x* × *f*, where *f* is the intra-train X-ray pulse frequency and Δ*x* is the midpoint separation (gap-to-gap spacing) [Fig. 3 ▸(*b*)].

There are two main differences in how the two methods measure jet velocity. First, the droplet PIV method can be used in a regular nozzle testing laboratory, while the jet explosion method requires access to an XFEL beam. On the other hand, the droplet PIV method measures the velocity of the droplets several millimetres downstream of the interaction point with the X-rays. Here, we compare the two methods with the same jetting parameters to verify whether the droplet velocity gives an accurate measure of the jet velocity at the interaction region.

**Figure 4:**
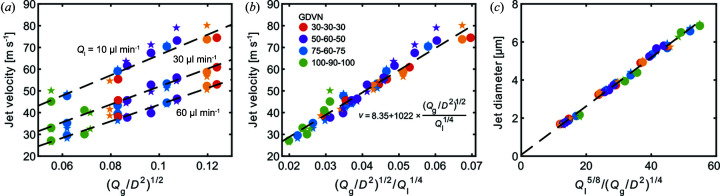
(*a*, *b*) Plots of the experimentally determined jet velocities for the four different GDVN types as a function of applied liquid and gas flow rates. Circles represent data from the jet explosion method, while stars denote velocities from the droplet PIV method. In panel (*b*) the broken line describes the jet velocity prediction formula *v*_jet_ = *a* + *b* × (*Q*_g_/*D*_g_^2^)^1/2^/(*Q*_l_)^1/4^, where the constants have the numerical values *a* = 8.35 and *b* = 1022 if *Q*_g_ is given in mg min^−1^, *D*_g_ in µm, *Q*_l_ in µl min^−1^ and *v*_jet_ in m s^−1^. (*c*) The corresponding jet diameters are calculated via *d*_jet_ = [4*Q*_l_/(π*v*)]^1/2^.

According to theory (Gañán-Calvo & Montanero, 2009 ▸; Beyerlein *et al.*, 2015 ▸), the jet velocity *v*_jet_ should follow the scaling law *v* ≃ Δ*P* ^1/2^, where Δ*P* is the pressure drop across the meniscus. Given the gas mass flow rate *Q*_g_ and the gas aperture diameter *D*, the pressure drop can be approximated by Δ*P* ≃ *Q*_g_/*D* ^2^. Notice how this expression is independent of the liquid flow rate *Q*_l_. The measured velocities are compared with the scaling law *v* ≃ (*Q*_g_/*D* ^2^)^1/2^ in Fig. 4 ▸(*a*) for four different GDVN geometries measured with varying *Q*_g_ and *Q*_l_. Although the scaling accurately predicts the velocity dependence of *Q*_g_ and *D*, we also observe a clear dependence on *Q*_l_ that is not predicted by Gañán-Calvo and Beyerlein. In addition, we can see that the droplet PIV and jet explosion methods give velocities that are similar within the variations expected from our analysis, but we cannot conclusively exclude a small amount of acceleration of the liquid between the X-ray interaction point and droplet formation region. In particular, we expect the droplet PIV method to produce larger variations in the velocity measurements due to the smaller Δ*x* compared with the jet explosion method [compare Δ*x* in Figs. 3 ▸(*b*) and 3 ▸(*c*)].

By including a *Q*_l_ dependence, we empirically obtained a remarkably accurate scaling *v* ≃ (*Q*_g_/*D* ^2^)^1/2^/(*Q*_l_)^1/4^ valid in the full parameter space of our measurements [Fig. 4 ▸(*b*)]. We would like to stress that we currently have no physical arguments for why the jet velocity should scale as 1/(*Q*_l_)^1/4^. Nevertheless, we believe that our scaling law can be used to predict the jet velocities within our parameter space for user experiments, as well as serving as a guide for nozzle designs in an extended parameter space. Finally, we show in Fig. 4 ▸(*c*) the jet diameters calculated from *d*_jet_ = [4*Q*_l_/(π*v*)]^1/2^. Using the scaling law found in Fig. 4 ▸(*b*), we see that the jet diameter is proportional to (*Q*_l_)^5/8^/(*Q*_g_/*D* ^2^)^1/4^ in the parameter space covered here.

Solely inferring the jet diameter from microscopic images (see the supporting information, Section S5) leads to a systematic overestimation of the jet diameter, which in turn implies much lower jet velocities. This misjudgement is not surprising, considering that the optical resolution of light microscopy systems is of the same order of magnitude as the jet diameter. Hence, the use of X-ray pulses, or the more accessible optical laser pulses, is indispensable for obtaining decisive jet velocities.

It is further worth noting that, in a vacuum, liquid jet acceleration is limited to the immediate vicinity of the nozzle, since gas flow decays quickly out of the orifice. In contrast, when jetting into an atmospheric sample chamber, the same nozzle configuration achieves jet velocities that are about five times greater (Knoška *et al.*, 2020 ▸).

### 3.2. Micromixing capabilities

**Figure 5:**
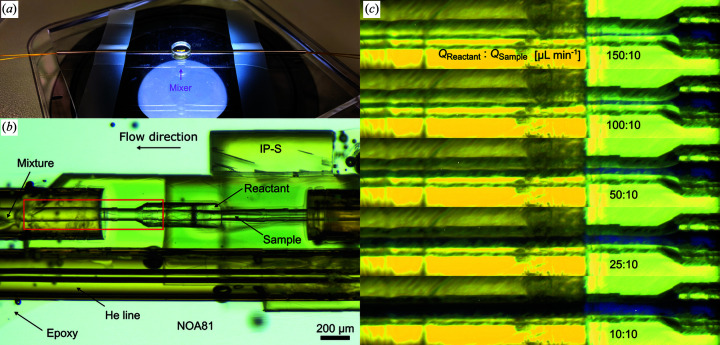
(*a*) A photograph of an assembled micromixer (type Y with 100 µm ID) before connection to a GDVN. (*b*) A microscopic image showing a detailed view of the mixing initiation area. (*c*) Magnified regions of the mixing channel–capillary interface, showing the 3D hydrodynamic flow focusing of a central ink stream (main channel) where the diluting water (entering from the side channel) runs at various flow rates. The mixed species is seamlessly transferred from the 2PP-3D printed part into the capillary extension.

The 3D concentric cones micromixers allow a modular assembly with different GDVNs and are available in two variations of 100 µm (Fig. 5 ▸) and 200 µm channel diameter (designs Y and Z, respectively). The choice of spacing between mixer and nozzle (*i.e.* the length of the bridging capillary) defines feasible time points in the range of several tens to hundreds of milliseconds. For accessing short time points, mixing-GDVNs, which have the nozzle tip directly connected to the short mixing channel, are on offer. They are available in two variations as well: design S has a long mixing channel of 2122.3 µm, while design V provides a 331.8 µm long channel. Both hybrids have mixing channels with a 100 µm ID and lead into a 75–60–75 µm GDVN (type B), capable of delivering *ca* 3–15 µm wide jets. In theory, even shorter time points (<3 ms) could be provided, but they would require operation parameters which are not relevant to the consideration of real-life SFX conditions/requirements, keeping in mind moderate sample consumption/dilution and compatibility with the EuXFEL’s high repetition rate.

In the supporting information (Section S4.4, gas flow evaluation) we discuss operational limits for these devices, such as compatible liquid/gas flow rates with respect to sample chamber applications (vacuum levels which have to be maintained for the specific detector on SPB/SFX that is under high voltage).

### 3.3. Mix-and-inject SFX

In general, 3D protein structure determination using SFX relies on the collection of a vast number of diffraction patterns (detector images in which X-rays hit a crystal) that can be assigned to specific unit cells, *i.e.* ‘indexed patterns’. The required number of indexed patterns for solving a structure depends considerably on the system but should be at least 5000 (Mehrabi *et al.*, 2021 ▸). However, for a high signal-to-noise ratio, the gathering of about 30000 indexed patterns is recommended, while the data quality scales in proportion to the square root of the number of patterns (Chavas *et al.*, 2015 ▸). For example, with an assumed indexing rate of all patterns of 50%, a hit rate (*i.e.* the ratio of X-ray pulses hitting a crystal to the available X-ray pulses) of 1% and an assumed number of 3520 pulses per second (*i.e.* 1.13 MHz mode), the estimated time it would take to obtain a complete data set is 5 min, but about 30 min for a highly redundant data set with better signal-to-noise ratio.

To verify the mixing capabilities under real-world SFX conditions, we investigated the diffraction of water/lysozyme mixtures with a type Z micromixer (200 µm ID channel diameter) with a total constriction length of 28.8 mm, connected to a 100–90–100 µm GDVN (type C). The utilized length was chosen by following the formulation in the literature (Calvey *et al.*, 2019 ▸).

The considerations for calculating this length were a protein-in-crystal concentration – *i.e.* average protein density (1350 g l^−1^) × protein content in the unit cell (usually around 50%) – of 891 g l^−1^, which (after being divided by the protein’s molecular weight, assuming 250 kg mol^−1^) equals 3.5 m*M*. Further, we assumed a reactant concentration of 30 m*M*, used the glucose diffusion coefficient of 6.0 × 10 m^2^ s^−1^ as an approximation, and considered a threshold concentration of 12.5% for achieving a probed time point of 500 ± 100 ms (which is on the higher end of usual time points).

During data collection, a wide range of flow rate ratios (FRR) were screened. Starting from a 10:1 (water:sample) FRR (11-fold dilution), the dilution was decreased with a 3:1 FRR (four-fold dilution). As documented in Table 3 ▸, we observed an expected FRR-dependent effect on the percentage of indexable hits: the lower the dilution, the higher the percentage. Note that 11-fold dilution did not make use of an inline filter (Table S1), which probably led to the arrival of more crystals in the beam. The diffraction data processing was performed using the *CrystFEL* package (Version. 0.9.1; White *et al.*, 2012 ▸). Peaks were detected and frames indexed using *peakfinder8* and *mosflm* (Leslie & Powell, 2007 ▸), respectively.

### 3.4. Electrospraying

The droplets produced by the 40 µm ID electrospray tips were characterized by a scanning mobility particle sizer (SMPS, TSI 3938) consisting of an electrostatic classifier (TSI 3082), a differential mobility analyser (DMA) (TSI 3081) and a condensation particle counter (TSI 3788). The electrosprayed droplets were neutralized right after nebulization by a soft X-ray source (Hamamatsu L9491) that creates a bipolar charge distribution by ionizing the sheath gas flow (Liu & Chen, 2014 ▸). By operating the electrospray with a sucrose solution with volume fraction *c* (here *c* = 0.05), the initial droplet diameter *D* could be determined from the DMA trace as *D* = *D*_suc_/*c* ^1/3^, where *D*_suc_ is the diameter of the sucrose particle after solvent evaporation (Chen *et al.*, 1995 ▸). For SPI applications, it is important to know the probability that a sample particle ends up inside a given droplet after nebulization, which is given by the volume-weighted size distribution. Thus, the values of *D* presented here will be the droplet diameter with the highest concentration as measured in the volume-weighted distribution.

**Figure 6:**
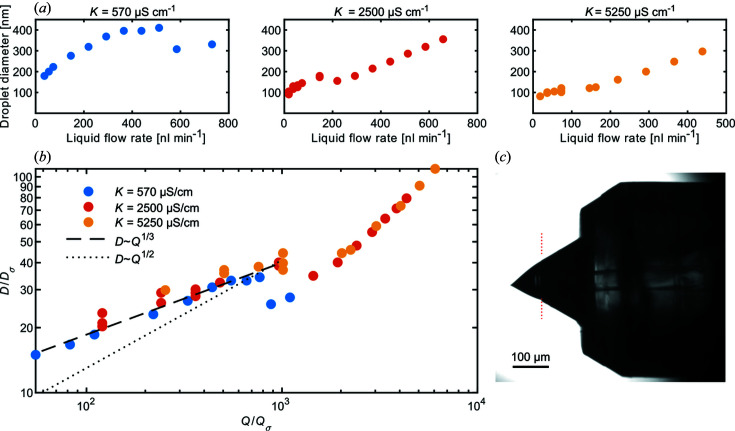
(*a*) Droplet diameters achieved as a function of applied liquid flow rate *Q* for different conductivities *K*. (*b*) In the dimensionless parametrization of the flow rate and droplet (Maißer *et al.*, 2013 ▸), the droplet diameters from the different conductivities fall on the same curve. (*c*) A side-view microscopy image (0.92 µm pixel size) of the 2PP-3D printed CCT. The 40 µm ID of the electrospray tip exactly matches the ID of the utilized fused silica capillary and the 30° angle at the tip is seamlessly transferred to the angle of the formed liquid cone. The tip–liquid interface is indicated with the dotted red line.

In Fig. 6 ▸(*a*), the *D* obtained at different liquid flow rates *Q* are shown for different solution conductivities *K*. In general, *D* increases with increasing *Q* and decreasing *K*. The *D* measured at different *K* values can be compared by introducing the dimensionless quantities *D*/*D_σ_* = *D*[*K* ^2^ρ/(σɛ_0_^2^)]^1/3^ and *Q*/*Q_σ_* = *QK*ρ/(σɛ_0_), where *K* is the electrical conductivity of the liquid, ρ its density, σ the surface tension and ɛ_0_ the vacuum permittivity (Maißer *et al.*, 2013 ▸). *D*/*D*_σ_ as a function of *Q*/*Q*_σ_ is shown in Fig. 6 ▸(*b*). In this representation, we see that *D* ≃ *Q* ^1/3^ when *Q*/*Q*_σ_ < 1000 (broken line), as was found by Chen & Pui (1997 ▸). The scaling of *D* ≃ *Q* ^1/2^ (dotted line) as observed by Maißer *et al.* (2013 ▸) does not provide an accurate match with our data. Above the ratio *Q*/*Q*_σ_ = 1000, the scaling becomes steeper and *D* increases more rapidly with *Q*, perhaps indicating a change in the force balance in the Taylor cone region (Gañán-Calvo, 2004 ▸).

## 4. Conclusions and outlook

Rapid prototyping using two-photon polymerization allows near-unlimited design flexibility and provides unmatched reproducibility to fabricate custom microfluidic sample delivery devices at high throughput. In combination with suitable injection/monitoring setups consisting of high-precision HPLCs and syringe pumps, gas pressure regulators, liquid/gas flow meters, high-speed video microscopes, microflow switching valves and stainless-steel sample reservoirs, they address a broad range of experimental conditions for SFX and SPI user experiments (low background, fast sample replenishment) on the SPB/SFX instrument.

Reliable liquid sample delivery systems on other scientific instruments can be achieved using similar solutions. Future designs will, for instance, enable Rayleigh jets for X-ray scattering and absorption spectroscopy experiments as pursued on the Femtosecond X-ray Experiments (FXE) instrument, while, on the Nano-sized Quantum Systems (NQS) endstation of the Small Quantum Systems (SQS) instrument, gas-phased samples such as protein complexes and a wide range of nanoclusters will be delivered efficiently by the 2PP-3D printed electrospray-based injectors.

## 6. Related literature

The following references, not cited in the main body of the paper, are cited in the supporting information: Bernardeschi *et al.* (2021 ▸); Doppler *et al.* (2022 ▸); Echelmeier *et al.* (2020 ▸); Haas (2000 ▸); Huang *et al.* (2020 ▸); Kotz *et al.* (2021 ▸); Lomb *et al.* (2012 ▸); Ristok *et al.* (2020 ▸); Schmid *et al.* (2019 ▸); Sikanen *et al.* (2012 ▸); Weierstall (2014 ▸); Weierstall *et al.* (2012 ▸).

## Supplementary Material

Supporting information: Sections S1 to S6; Figures S1 to S11; Tables S1 to S4. DOI: 10.1107/S1600577521013370/gb5122sup1.pdf

## Figures and Tables

**Table 1:** Overview of X-ray parameters for various example settings on the SPB/SFX instrument The pulse energies in settings 2–4 are comparable as the accelerator is usually running at 2.26 MHz and electron bunches are delivered interleaved to SASE1 and SASE3 (*i.e.* maximum 1.13 MHz delivered to each of SASE1 and SASE3). The number of X-ray pulses per 10 Hz train can be varied, leading to different intra-train repetition rates.

	Example values			
Parameter	1	2	3	4
Pulse duration (fs)	*ca* 100			
Photon energy (keV)	3–16	9.3	9.3	9.3
Pulse energy (mJ)	4	1–2	1–2	1–2
Beam size (µm)	*ca* 3			
Photon flux (photons pulse^−1^)	1.0 × 10^12^			
Photon flux (photons s^−1^)	2.7 × 10^16^	1.4 × 10^16^	6.8 × 10^15^	2.0 × 10^15^
Brilliance [photons s^−1^ mm^−2^ mrad^−2^ (0.1% bandwidth)^−1^]	5 × 10^33^			
No. of pulses per train	2700	1350	352	202
Time between two pulses (ns)	221.6	443.3	886.5	1773.0
Duration of pulse train (µs)	598	598	312	358
Intra-train rep. rate of pulses (MHz)	4.51	2.26	1.13	0.564
Time between two trains (ms)	100			
Repetition rate of trains (Hz)	10			
No. of pulses per second	27000	13500	3520	2020
Jet gap-to-gap spacing (µm)	∼11	∼22	44†	∼88
Jet velocity (m s^−1^)			49.6	

† Observed spacing between two gaps along the irradiated liquid jet (displacement between two pulses) in 1.13 MHz mode.

**Table 2:** List of microchannel design parameters for the 2PP-3D printed liquid sample injection devices with an indication of required liquid flow rates (in µl min^−1^) for stable operation and compatible pulse operation modes (intra-train pulse repetition rates) The denoted parameters for GDVNs describe the liquid orifice diameter *D*_liquid_, the gas orifice diameter *D*_gas_ and the distance between these two orifices *H*_liquid-gas_. Mixing devices are further described by the diameter of the mixing channel *D*_channel_, the diameter of the main channel (sample inlet) *D*_MC_, the diameter of the side channel (reactant inlet) at the mixing initiation region *D*_SC_ and the mixing channel length *L*_channel_. HVEs and CCTs for electrospray purposes are further described with their wall thickness at the tip *z*_wall_ and the angle of the cone. This list and the referenced designs can be found online in our corresponding GitHub repository, https://github.com/flmiot/EuXFEL-designs.

Device type	Design parameters	*Q*_liquid_ (µl min^−1^)	Pulse mode	Design name
GDVN	*D*_liquid_–*D*_gas_–*H*_liquid-gas_ (µm)			
	30–30–30	≥5	≤2.3 MHz	JKMH_5†, MVED_F
	50–60–60	≥8	≤2.3 MHz	JKMH_6†
	75–60–75	≥8	≤1.1 MHz	MVED_B
	100–90–100	≥10	≤0.5 MHz	MVED_C
	150–100–150	≥75	≤0.14 MHz	MVED_D
	180–145–180	≥100	≤0.14 MHz	MVED_E
Micromixer	*D*_channel_–*D*_MC_–*D*_SC_–*L*_channel_ (µm)			
	100–75–180–300	≥10	Depends on GDVN	MVED_Y‡
	200–100–231.7–300	≥10		MVED_Z‡
	200– (200/2)–(200/2)–950	≥10		JKMH_10† §
	100–(100/2)–(100/2)–950	≥10		JKMH_10H† §
Mixing-GDVN	*D*_channel_–*D*_MC_–*D*_SC_–*L*_channel_–*D*_liquid_–*D*_gas_–*H*_liquid-gas_ (µm)			
	100–75–180–2122.3–75–60–75	≥10	≤1.1 MHz	MVED_S
	100–75–180–331.8–75–60–75	≥10	≤1.1 MHz	MVED_V
DFFN	100–75–96–445.6–75–70–70	≥5 + ≥15 (EtOH)	≤1.1 MHz	JKMH_8†
HVE	*D*_liquid_–*D*_gas_–*H*_liquid-gas_–*z*_wall_–α (µm, °)			
	50–365–600–20–9	≥0.3	10 Hz	MV_K
	75–365–600–20–9	≥0.3	10 Hz	MV_L
	100–365–600–20–9	≥0.3	10 Hz	MV_T
Mixing-HVE	*D*_channel_–*D*_MC_–*D*_SC_–*L*_channel_–*D*_liquid_–*D*_gas_–*H*_liquid-gas_–*z*_wall_–α (µm, °)			
	231.7–100–231.7–2570–100–345–600–20–9	0.3–3	10 Hz	MV_I
	231.7–100–231.7–2570–75–345–600–32.5–9	0.3–3	10 Hz	MV_J
CCT	*D*_channel_–*L*_channel_–*z*_wall_–α (µm, °)			
	40–200–50–30	0.02–0.8	≤4.5 MHz	MV_W
	10–200–10–20	0.02–0.8	≤4.5 MHz	MV_X

† These designs were previously discussed elsewhere (Knoška *et al.*, 2020 ▸).

‡ Concentric cones.

§ Kenics.

**Table 3:** SFX data collection and refinement statistics The entire peak search and index processing was performed identically across all data sets. The hit rates for dilution set 8 are unusually high. Under normal experimental conditions, each data set would be optimized independently. In this case, hit-finding is treated identically and the higher hit rates are probably due to the changes in jetting conditions and subsequent changes in detector background. More accurate values can be seen in the number of indexed crystals. The respective data quality statistics fall within typically acceptable values consistent with previous experiments.

	Dilution†				
Parameter	11	8	6	5	4
Total number of frames	3645696	3038080	1822848	608020	634684
Total number of frames with hits	206283	3037676	1155073	75838	36550
Hit rate (%)	5.66	99.99	63.37	12.47	5.76
Number of indexed crystals	80254	5 605	10 214	1 695	5 046
Proportion of indexable hits (%)	38.9	0.18	0.88	2.24	13.81
Unit-cell lengths (Å)	79.4, 79.5, 38.6				
Unit-cell angles (°)	90, 90, 90				
Resolution (Å)	32.1–2.0 (2.07–2.0)				
*R*_split_ (%)	6.89 (14.79)	24.11 (53.17)	20.24 (36.81)	44.05 (93.68)	28.88 (72.36)
CC_1/2_ (%)	99.20 (95.06)	91.12 (64.27)	90.04 (71.85)	74.66 (25.53)	87.99 (52.61)
CC* (%)	99.80 (98.73)	97.65 (88.46)	97.34 (91.44)	92.46 (63.78)	96.75 (83.04)
Signal-to-noise ratio	13.24 (7.14)	3.94 (1.96)	4.98 (2.89)	2.23 (1.03)	3.07 (1.26)
Completeness (%)	100 (100)	100 (100)	100 (100)	99.76 (97.82)	99.98 (99.81)

† Details of the respective mixing/jetting parameters can be found in the supporting information, Section S4.2.
