# Supplementary material for: 3D printed devices and infrastructure for liquid sample delivery at the European XFEL
Source: J Synchrotron Radiat. 2022 Feb 15;29(Pt 2):331–46. doi: 10.1107/S1600577521013370 (PMC8900844; doi:10.1107/S1600577521013370)
Supplement: Supplementary file 1 [file s-29-00331-sup1.pdf]

JOURNAL OF SYNCHROTRON RADIATION

Volume 29 (2022)

Supporting information for article:

3D printed devices and infrastructure for liquid sample delivery at the European XFEL

Mohammad Vakili, Johan Bielecki, Juraj Knoška, Florian Otte, Huijong Han, Marco Kloos, Robin Schubert, Elisa Delmas, Grant Mills, Raphael de Wijn, Romain Letrun, Simon Dold, Richard Bean, Adam Round, Yoonhee Kim, Frederico A. Lima, Katerina Dörner, Joana Valerio, Michael Heymann, Adrian P. Mancuso and Joachim Schulz

S1. Instrumentation

Optical microscopy images were taken with an Olympus SZX16 equipped with a 9-megapixel UC90 digital camera.

Fluorescence imaging was realized by a Ni-E microscope equipped with a DS-Qi2 camera, a 4× or 10× objective (all Nikon), a pE-4000 illumination system (CoolLED) for providing visible light and a single-band filter set (Semrock, YFP-2427B-000). The filter set was composed of an excitation filter that restricts the range of wavelengths illuminating the sample to 500/24 nm, a 520 nm-edge dichroic mirror that reflects excitation light down to the sample while transmitting light emitted from the sample up towards camera, and an emission filter that restricts the range of wavelengths reaching the camera to 542/27 nm.

High-speed images were taken either with a Photron Mini AX camera equipped with a 20× Plan Apo objective (Mitutoyo MY20X-804, 0.42 NA).

Flow experiments were carried out using LC-20AD XR high-pressure liquid chromatography systems (Shimadzu). HVE injection used gas-tight syringes by Hamilton controlled on a Nemesys 290N syringe pump (Cetoni) and Legato 130 (KD Scientific Inc.).

S2. Device fabrication

The designs of the described injection devices and hardware parts can be found under https://github.com/flmiot/EuXFEL-designs.

S2.1. Slicing, printing, developing

The microfabrication followed general guidelines detailed elsewhere.(Knoška *et al.*, 2020) 3D geometries were designed in AutoCAD (Autodesk) or NX (Siemens) and exported as STL formats (STEP214 files were exported via Autodesk Inventor). Using DeScribe (Nanoscribe), the STL-based 3D designs were converted to print-job instructions (GWL). To ensure highest possible structural stability, the devices were printed in solid volumes. For fast printing times, slicing distances of 1–2 µm (GDVNs: 1 µm, mixer and HVEs: 2 µm), hatching distances of 0.7 µm (0.5 µm for GDVNs and devices aimed for optical microscopy experiments), and block sizes of 285/285/299 µm (*x*/*y*/*z*) with 15° block shear angles, 2 µm block overlaps and 1 µm layer overlaps were chosen.

The devices were then printed with the Nanoscribe Photonic Professional GT in upwards direction (+*z*) with alternating hatch lines and in the dip-in mode. Preferably, the IP-S photoresist was used (see S6). The resist was deposited onto an indium tin oxide (ITO) coated glass slide. The printer was equipped with an 25x objective lens (Zeiss), the laser power was tuned to 100% (*i.e.* 156 mW exiting

the laser source, 70 mW arriving at the 25x objective), and print speeds were 100 000 µm s^{-1}. Under these parameters, it took *ca.* 45 min to print one IP-S GDVN. For different materials, different slicing options, laser powers, and scan speeds were applied. Table S1 provides details on utilized slicing/hatching distances, printing procedures, and post-printing processes for various device types.

Table S1 Details on procedures for device fabrication and assembly of 2PP–3D printed sample delivery devices. The slicing and hatching distances are given in µm, laser power in %, and scan speed in µm s^{-1}.

Material	Device	Device fabrication					Assembling	
		Slicing	Hatching	Laser power	Scan speed	Development	Gluing	Glue aging[3)]
IP-S	GDVN	1	0.5	100	100 000	PGMEA, overnight	Epoxy, 12 h at RT	NA
	DFFN[1)]	1	0.5	100	100 000	PGMEA, overnight	NOA68, 30 min UV	12 h at 50°C
	HVE	2	0.7	100	100 000	PGMEA, overnight	Epoxy, 12 h at RT	NA
	Micromixer[2)]	2	0.5	100	100 000	PGMEA, overnight	1: Epoxy, 12 h at RT<br>2: NOA81, 30 min UV	12 h at 50°C
	CCT	1	0.5	70	100 000	PGMEA, overnight	Epoxy, 12 h at RT	NA
OrmoComp[4)]	GDVN	1	0.5	75	50 000	OrmoDev, overnight	Epoxy, 12 h at RT	NA
	Micromixer	2	0.5	75	50 000	OrmoDev, overnight	1: Epoxy, 12 h at RT<br>2: NOA81, 30 min UV	12 h at 50°C

1) Epoxy glue has a low chemical resistance towards ethanol. Therefore, capillary tips can be burned off (flame-assisted removal of the *ca.* 20 µm thick polyimide coating) before insertion into a DFFN device and secured with the viscous NOA68.

2) Gluing of micromixer proceeds in two steps as described in S2.2.1.

3) As recommended by the manufacturers, the adhesion can be improved with age. Full cure of Devcon epoxy is reached after 12 h at RT (should not be treated above 90 °C). Loctite 3450: 12 h at 80 °C (or 7 days at RT). NOA: Optimum adhesion is obtained by aging at 50 °C for 12 hours (or 7 days at RT).

4) After development, OrmoComp device were UV-cured for 60 min followed by a hard bake at 150 °C for 3 h.

After printing, the glass slide with the cured photoresist was put into a beaker filled with *ca.* 10 mL of propylene glycol methyl ether acetate (PGMEA, Merck) for one to two days to dissolve uncured parts (development). Then, the devices were transferred into a beaker with isopropanol (VWR chemicals), where they were allowed to sit for 30 min before being transferred into another beaker with fresh isopropanol. A few hours prior to assembly they were transferred into another isopropanol-beaker before being pipetted onto a cleanroom cloth where they were dried in atmosphere.

S2.2. Assembling

Device assembly proceeded on a clean polydimethylsiloxane (PDMS, DowSil) sheet (which provides a transparent substrate with high grip) which was placed onto a moveable Petri disk. Another piece of PDMS was placed on top of the device to fix the device in place. Then, under an optical microscope, one (HVEs/CCTs), two (GDVNs) or three (mixing-GDVNs/DFFNs) fused silica capillaries (Polymicro, 360 µm OD) were inserted into the fluid access ports and glued in place using 5 min epoxy glue (Devcon).

The capillaries needed to be at least 2 m long to bridge the distance between the SPB/SFX interaction region upstream (IRU) and the switching valves for the liquids (Rheodyne, MX Series II) on top of the sample chamber. For HVEs and CCTs, capillary lengths of 80 and 400 mm, respectively, were cut down. One end of the capillary was ground at an attack angle of 90° (Allied, MultiPrep 8 in) and subsequently sonicated in water before connection in order to enable a clean and smooth capillary edge for seamless transitions and to avoid sample agglomeration at the capillary–device intersection. For the GDVNs, the capillary–nozzle assembly was then fiddled through to a hollow stainless-steel tubing of 1/16 in OD (IDEX U-115 with 0.03 in ID) and glued between the nozzle material and steel. Including the time to prepare the required consumables and instrumentation, it took in general, *ca.* 1 h to assemble/glue a set of three such nozzles, and the entire expense of one deployable nozzle amounted to *ca.* €50 (without tax).

With the stiff steel tubing, a tight connection with #10-32 UNF fittings (PEEK, IDEX F333N) could be achieved to stainless steel “nozzle holders” containing an external M9×1 mm thread (Weierstall, 2014), which further allowed connection to the liquid jet injection hardware at the SPB/SFX instrument.(Schulz *et al.*, 2019) Due to the additional inlet capillary, mixer assemblies required 0.046 in ID steel tubings (IDEX, U-145).

S2.2.1. Assembly specific to mixing-GDVNs

To facilitate a platform for assembling mixing devices, replica moulding strategies were pursued (Fig. S1). An aluminium master, machined by CNC milling, was placed into a tin foil-covered beaker. This template contained an 8.47 mm wide and centred cylinder with two adjacent 1/16 in wide ligaments. Its shape was replicated using PDMS. After cross-linking at 80 °C for 2 h, the PDMS replica was peeled off the Al master, and two steel tubings (1/16 in OD, 0.046 in ID) were inserted into the now-obtained PDMS through holes to later surround the fluid lines. In the PDMS manifold's centre, the micromixer, elevated by the *mixer pedestal* (MV_Q, which brings access ports to the same height as the centre of the steel tubing's ID), was placed with a tweezer and then connected to the fused silica capillaries entering via steel tubing. Here, 3 capillaries (sample, reactant, gas) entered the device, while 2 capillaries (reaction mixture, continuation of gas line) exited the device. Then, sealing occurred by adding epoxy glue to the device's access ports and dispensing it in a way that connected IP-S material with the steel. The choice of exit steel tubing (length may be as short as 15 mm) should be slightly shorter than the exit capillary length. After 12 h of epoxy glue curing at room temperature, 192 µL of NOA81 (Norland Products, Inc.) glue were pipetted into the circular opening to fully enclose the mixer. After the NOA was UV-cured for 30 min, the overall mechanical stability was significantly increased and, due to the straight line of two immobile tubings (with the NOA81 glue chip as the joint in between), the insertion and alignment at the beamline instrument was facilitated. Finally, the exit liquid and gas capillaries were inserted into a GDVN module and glued with epoxy. The entire device was then baked at 50 °C for 12 h for optimal glue aging.

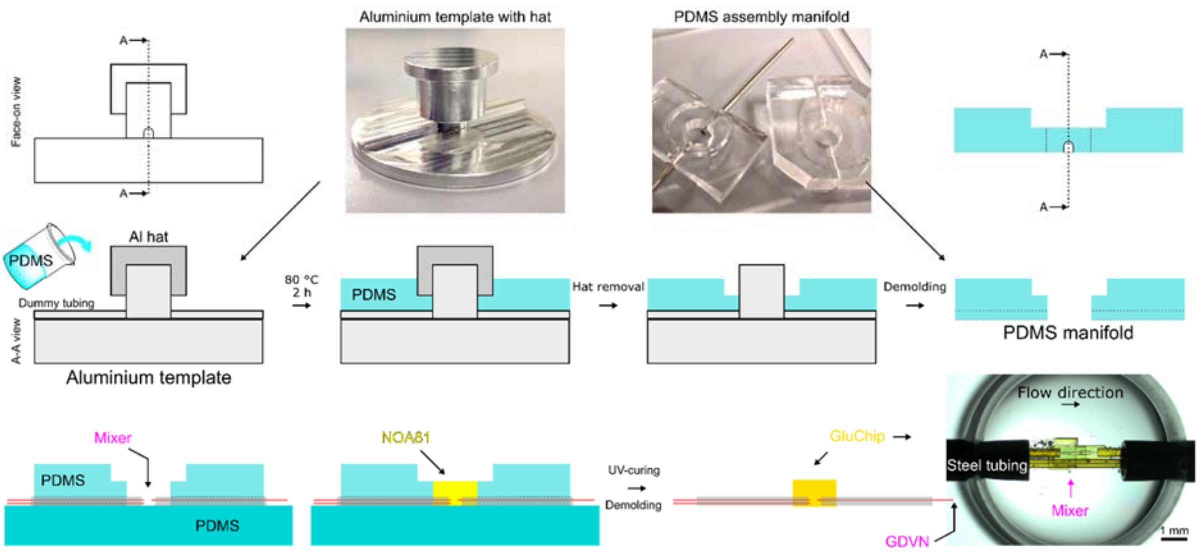

Figure S1 Soft lithographic fabrication sequence for the PDMS assembly manifold with visualization of the micromixer gluing procedure. The helium line is omitted from this schematic depiction. A photo of the final NOA81-protected "GluChip" with the encapsulated IP-S micromixer is shown at the lower-right corner.

S2.2.2. Assembly specific to HVE injectors

A capillary with 100 µm ID was cut down to *ca.* 80 mm in length and inserted into the access port of the 2PP–3D printed tip (Fig. S2 B). The epoxy glue was carefully attached (using another short capillary or a thin, bendable wire) onto the inserted capillary at the IP-S–capillary interface. After curing, the capillary was fiddled through a 25 mm long steel tubing (modified from IDEX U-115, 0.03 in ID, 1/16 in OD). It was important that the amount of previously deposited glue does not exceed the total thickness of the 2PP–3D printed part (dotted line in Fig. S2 C). Otherwise, the metal tubing could not be imposed on the IP-S–capillary assembly later. Next, the glue was attached onto the device's neck at the steel–IP-S interface (Fig. S2 D–F). Again, this had to be done carefully and multiple times, as some glue slid into the metal sleeve.

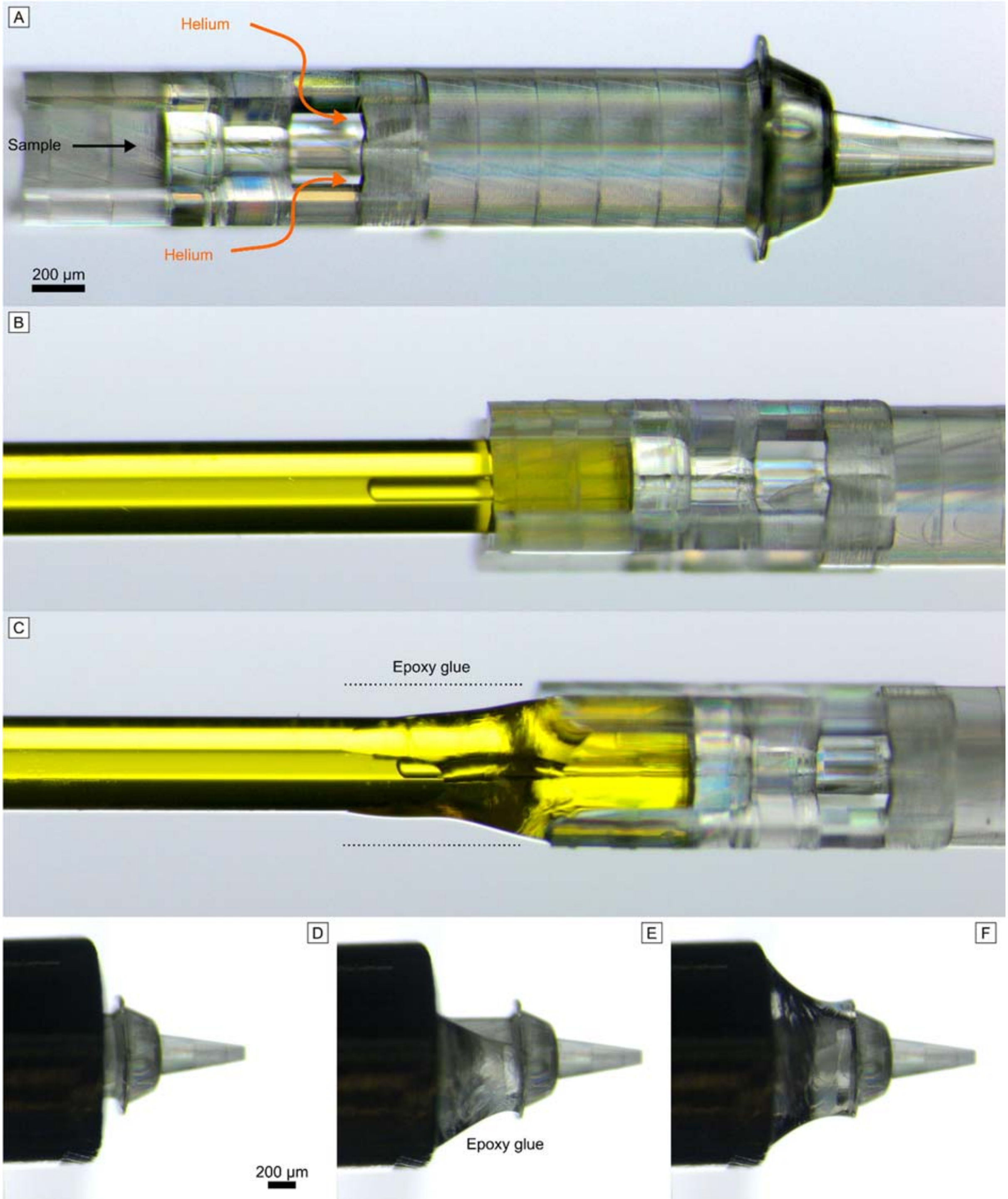

Figure S2 (A) Optical microscopy image of a 2PP–3D printed HVE device (type T). (B–F) Depiction of the gluing procedure towards an HVE injector tip with 1/16 in OD tubing compatibility.

A thus prepared injector tip (*i.e.* IP-S tip glued to a capillary and steel tubing) was secured with a tweezer at the tubing. Then, a steel nut (IDEX F-354, groove must face the nozzle tip) was carefully slipped over the tubing from the capillary side, followed by a coned ferrule (IDEX, LT-135) in such a way that the metal part faced the nut (Fig. S3 A). The steel tubing had to look out of the ferrule *ca.*

2 mm before screwing the nut into a T-shaped PEEK union (IDEX P-727-01, which had three female #10-32 UNF threads) using a custom wrench tool (*HVE_Tool*, which accessed the 1.5 mm wide dents of the nut; Fig. S3 B). Next, a column coupler (IDEX, U-281) was screwed tightly into the opposite port of the T-union, and the capillary was shortened so that *ca.* 1 mm (final capillary length *ca.* 50 mm) were sticking out of the column coupler (Fig. S3 C). Then, at the free end of the column coupler, a P-627 union (IDEX) was attached. This union had a female #10-32 UNF (coned) to female ¼-28 UNF (flat-bottom) thread configuration which allowed connectivity to Hamilton glass syringes (Model 1001 with ChemSeal ¼-28 UNF termination; Fig. S3 D). For the gas line, a 1/16 in OD PEEK tubing (125 µm ID) was connected to the free port of the T-union using a #10-32 UNF (IDEX, F-333NX) fitting (Fig. S4 C).

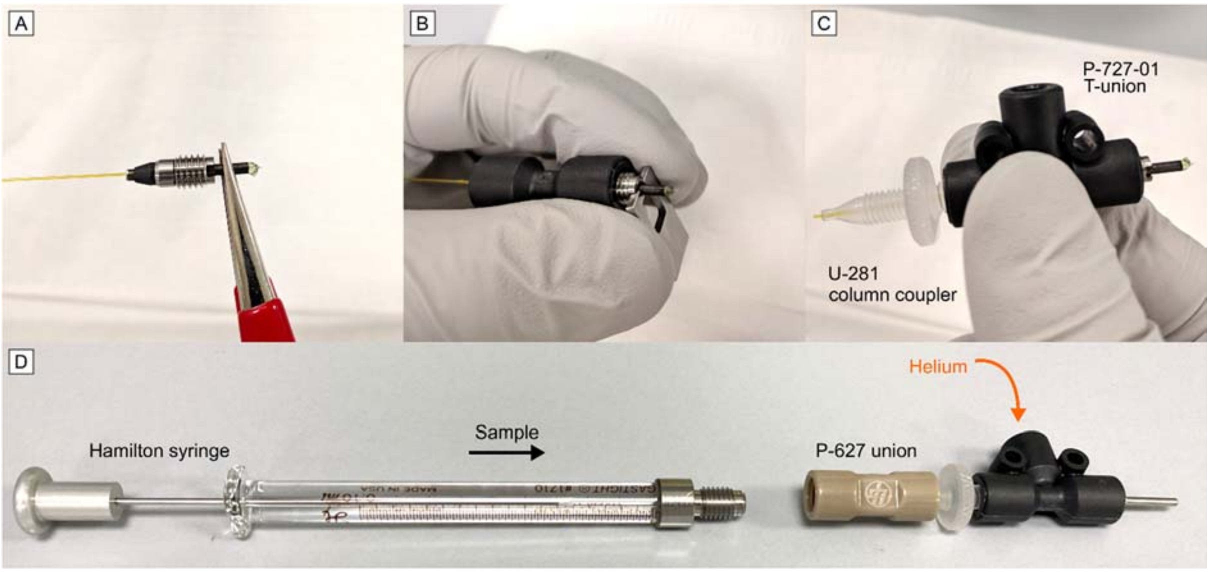

Figure S3 An HVE injector tip is assembled to facilitate an HVE-syringe injector.

Fig. S4 shows a photo of an HVE-syringe injector assembly in action. The use of wider-ID capillaries allowed for much longer capillary lengths than the 50 mm shown in Fig. S3. For instance, 250 µm ID capillaries can be used for longer separations between injector tip and sample-feeding syringe pump, which then allows high-viscous LCP injection at the interaction region downstream (IRD; see Fig. S6).

Besides the syringe-pump approach, conventional HVE injector systems with integrated reservoirs containing sample volumes of 20–120 µL (Weierstall *et al.*, 2014) and 400 µL (Doak *et al.*, in preparation), respectively, are available. They contain an externally positioned 12.07×5.33 (ID×cord

diameter, FKM 80A) O-ring on a steel rod to allow experiments both in vacuum and in the atmosphere. Here, the O-ring to injector tip distance is *ca.* 10 cm.

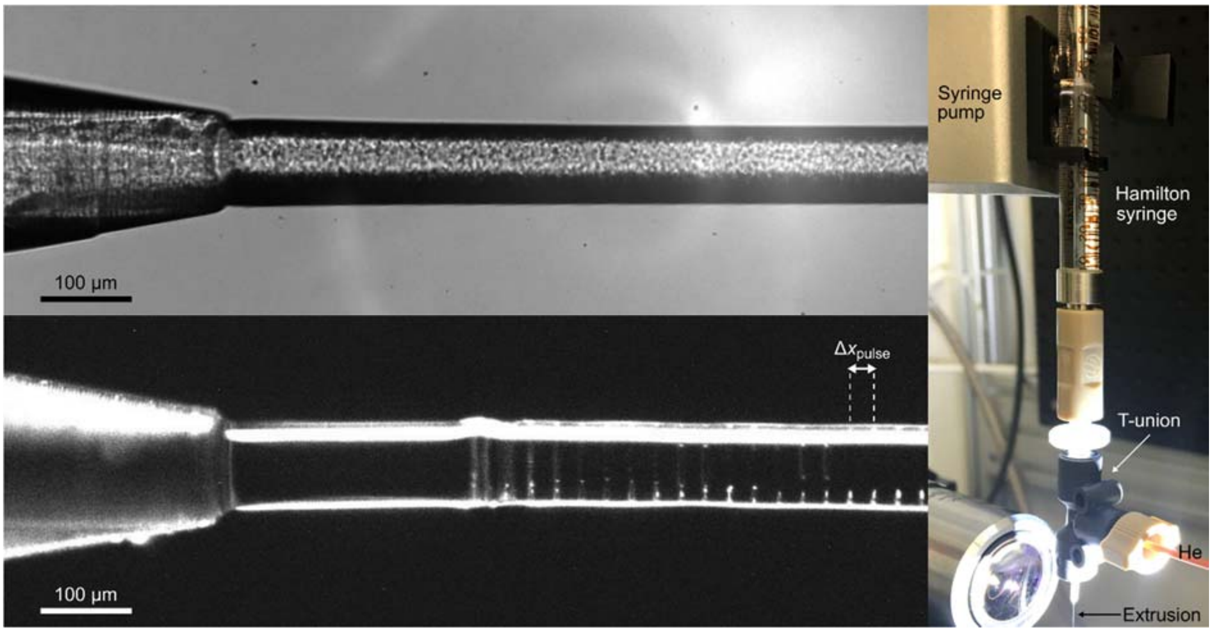

Figure S4 (A) Silicon grease (Synco Lube, DE 92003) being extruded through a 75 µm ID HVE tip using a liquid flow rate of 0.5 µL min^{-1} and a He pressure of 15 psi (11 mg min^{-1}). (B) 100 µm wide LCP stream (Q_l = 0.5 µL min^{-1}, Q_g = 11 mg min^{-1}) being probed by X-ray pulses arriving at 10 Hz. The X-ray interaction created visible marks along the sample stream. The distance between these ladder steps (*ca.* 30 µm) multiplied with the repetition rate results in a stream velocity of 0.3 mm s^{-1}. (C) Photo of a compact lab-based injection set-up that used standard fluid connection parts, gas-tight glass syringes as expendable sample reservoirs, and a high-precision syringe pump.

S3. Jet velocity experiments

The liquid jet velocity experiments (internal use proposal leading to experiment 2725) were conducted at SPB/SFX with X-rays (photon energy of 9.3 keV, beam size *ca.* 4×6 µm^2) arriving in 10 Hz trains with 30 pulses per train and an intra-train pulse repetition rate of 0.564 MHz. For the illumination, a visible light laser with a wavelength of 532 nm, a pulse length of 5 ns, and a pulse repetition rate of 10 Hz was used. The spot size was *ca.* 10 mm. Using dual-pulse laser illumination, the droplet region (2 mm downstream from the nozzle tip) was captured using variable delay times between the two pulses (between 70 and 500 ns) in order to determine the velocity of single droplets.

The side view microscope was a Zyla 5.5 sCMOS equipped with a 10× objective (Mitutoyo MY10X-803, 0.28 NA) leading to a pixel size of 0.65 μm and a theoretical Abbe's optical resolution of 0.95 μm. An experimental resolution of 1.38 μm was revealed by imaging an USAF resolution target.

In each data collection run, 600 side microscope images were captured within one minute (10 Hz collection rate) with each run being dedicated to a single nozzle/liquid flow rate/gas flow rate combination.

S4. SFX experiments at the SPB/SFX instrument

S4.1. Lysozyme crystal preparation

For the mix-and-inject experiment using 2PP–3D printed mixing-GDVNs, lysozyme crystals with 2–3 μm in diameter in 10% NaCl and 50 mM NaOAc (pH 3.5) were used. The crystals were prepared by rapid vortexing of a 1:2 ratio of lysozyme (100 mg mL^{-1} in 50 mM NaOAc (pH 3.5) and crystallization solution (0.1 M NaOAc (pH 3.5), 5% PEG6000, 3.2 M NaCl) at 10 °C. After crystallization, the liquid was exchanged to a storage/injection buffer (50 mM NaOAc (pH 3.5), 10% NaCl), and the crystal density was adjusted to be 22–25% after settlement. Before loading the sample reservoir, the crystal slurry was run through a Nylon mesh gravity filter (20 μm wide meshes, CellTrics) to remove large particles. Details on the utilized reservoir and crystal anti-settling device can be found elsewhere.(Lomb *et al.*, 2012)

S4.2. Beam and jetting parameters

The lysozyme jets (SPB/SFX commissioning during the setup days prior to experiment 2695) were probed with 9.3 keV X-rays with a beam size of 4.8×4.6 μm^2 FWHM (3.5×3.5 μm^2 during leg 1), arriving in 10 Hz trains with 202 pulses per train (180 during leg 1), and an intra-train pulse repetition rate of 0.564 MHz. The sample-to-AGIPD distance was 121.8 mm. Details on applied flow rate ratios can be found in Table S2.

Table S2 Mixing and jetting parameters for the SFX experiment. The flow velocity and retention time before entering the GDVN were determined by the channel length and ID (200 µm).

Channel length [µm]	Q_l [µL min^{-1}]			Flow rate ratio (FRR)	Dilution	Flow velocity [mm s^{-1}]	Retention time [ms]	Sample[2)]
	SC	MC	total					
28782.5	70	7	77	10:1	11.0[1)]	81.7	704.6	A
	35	7	42	5:1	6.0	44.6	1291.8	A
	65	15	80	4.33:1	5.33	84.8	678.2	B
	70	10	80	7:1	8.0	84.8	678.2	B
	60	20	80	3:1	4.0	84.8	678.2	B

1) Using jet explosion method, a jet velocity of 36.2 m s^{-1} was determined for this jet, which corresponded to a jet diameter of *ca.* 11.3 µm. Our prediction formula (Fig. 4(*b*)) revealed 30.5 m s^{-1} and 7.5 µm, respectively.

2) Sample A: Lysozyme in 10% NaCl, 2–3 µm, 22% density, 20 µm gravity filtered, no inline filter, 125 µm ID tubing. Sample B: Lysozyme in 10% NaCl 50 mM NaOAc, 2–3 µm, 22% density, 20 µm gravity filtered, inline filter with 40 µm^2 wide pores, 64 µm ID tubing.

S4.3. Injection hardware

The injector rods utilized at the interaction region upstream (IRU) of the SPB/SFX instrument (Fig. S5) are stainless steel hollow cylinders with a length of 1.2 m, and outer diameter (OD) of 12.7 mm, and inner diameters (ID) of 6.35 and 9.35 mm, respectively.(Schulz *et al.*, 2019) Two versions of the rod are currently in operation. They were developed by the EuXFEL user community and primarily differ in their connectivity at the bottom end. The “Heidelberg rod”, which is based on previous systems from contributors from the Max Planck Institute for Medical Research in Heidelberg,(Weierstall *et al.*, 2012) is the main rod for GDVN operation. It offers a M9×1 mm internal thread at its bottom for attachment of the nozzle holder and a knob for gripping and rotating the rod on its upper end. This rod has a 23 mm OD O-ring (12.07×5.33 mm, ID×cord diameter, FKM 80A) fixed directly at the rod (*ca.* 50 mm away from the bottom end) and is inserted into a 22 mm ID funnel receptor sitting on top of the catcher shroud.

To accept wider and longer liquid injection devices, such as multi-capillary mixing injectors, segmented flow generators (Echelmeier *et al.*, 2020) or co-flow injectors (Doppler *et al.*, 2022), the catcher receptor funnel for the Heidelberg rod is replaced with an alternative receptor funnel of 32 mm ID (or 35 mm). With this setup, devices up to 29 mm width can be placed inside the catcher, while being connected to the “Cornell rod” which was provided by contributors form the School of Applied and Engineering Physics at Cornell University (Calvey *et al.*, 2019). The Cornell rod has a

flat flange with an OD of *ca.* 20.4 mm and a height of 5.2 mm that is welded to the bottom of the rod. It provides an internal groove for housing a 7×2 mm or 14×2 mm O-ring for sealing. Via this flange, various custom adapters containing a M22×1.5 mm internal thread can be connected at the bottom end (Fig. S5, right). The vacuum seal at the catcher's funnel receptor is then enabled by another external O-ring sitting directly on the adapter.

With the Heidelberg rod, the distance from the rod's O-ring to the nozzle tip is *ca.* 130 mm. Using the Cornell rod, where the attached adapter provides a 33 mm OD O-ring, the distance from O-ring to nozzle tip can either be 110–135 mm (short funnel) or 110–180 mm (long funnel).

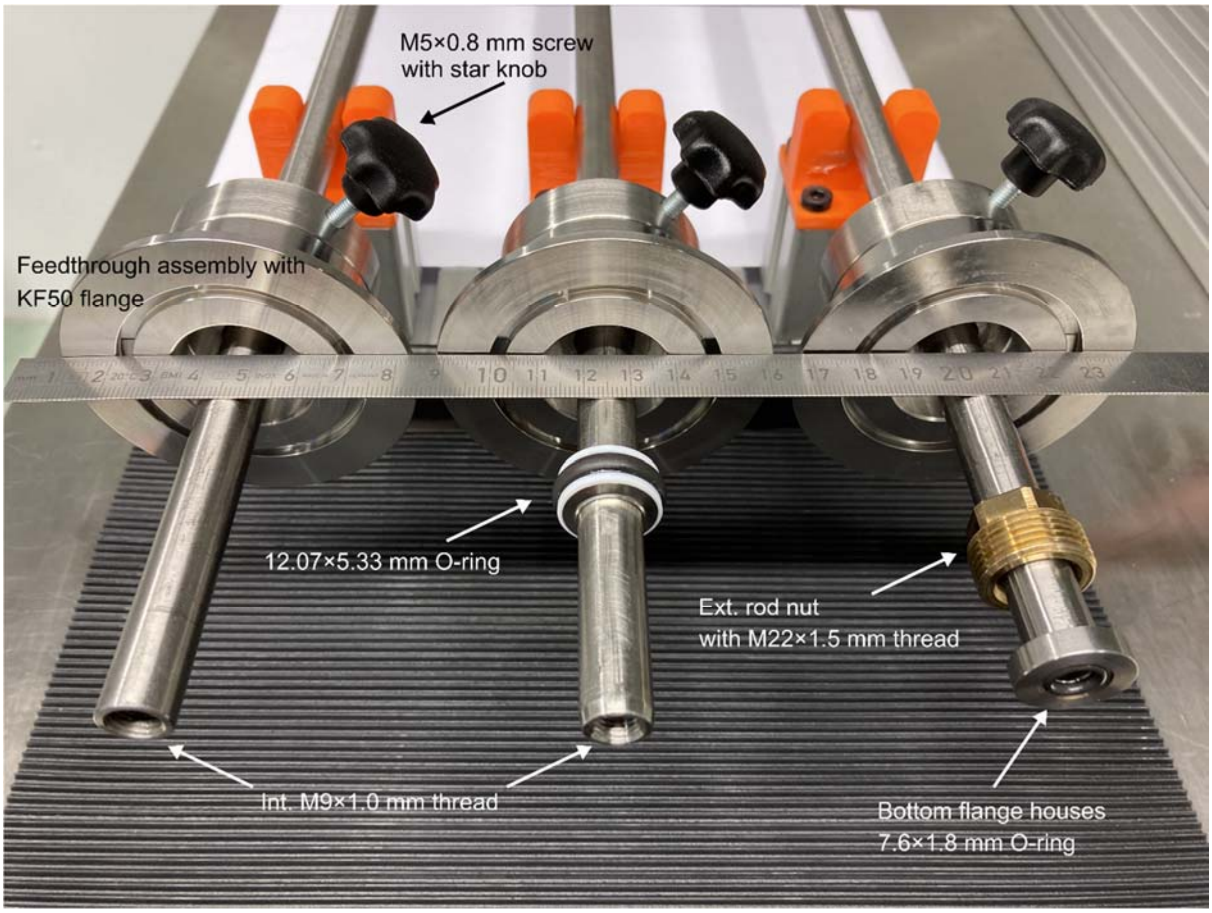

Figure S5 Photos of the injection rod no. II (left) for HVE injectors at the IRU, no. 14 (middle) for regular GDVN operation (Heidelberg rod), and no. 4 (right) for Cornell-type mixing injectors with description of their main bottom attachment features. Details on the rods can be found in Table S3.

For in-air HVE experiments at the interaction region downstream (IRD), similar rods with shorter lengths in combination with a wider bore funnel receptor (35 mm ID) screwed onto a modified, helium-purged, 6-way cube (ThorLabs, LC6W), are used (Fig. S6).

The available rods for device insertion into the SPB/SFX X-ray interaction regions are listed in Table S3. The chart provides details on their dimensions and adapter specifications. To adapt for different user needs and to further improve sample delivery and device exchange efficiency, modifications to the setups are expected.

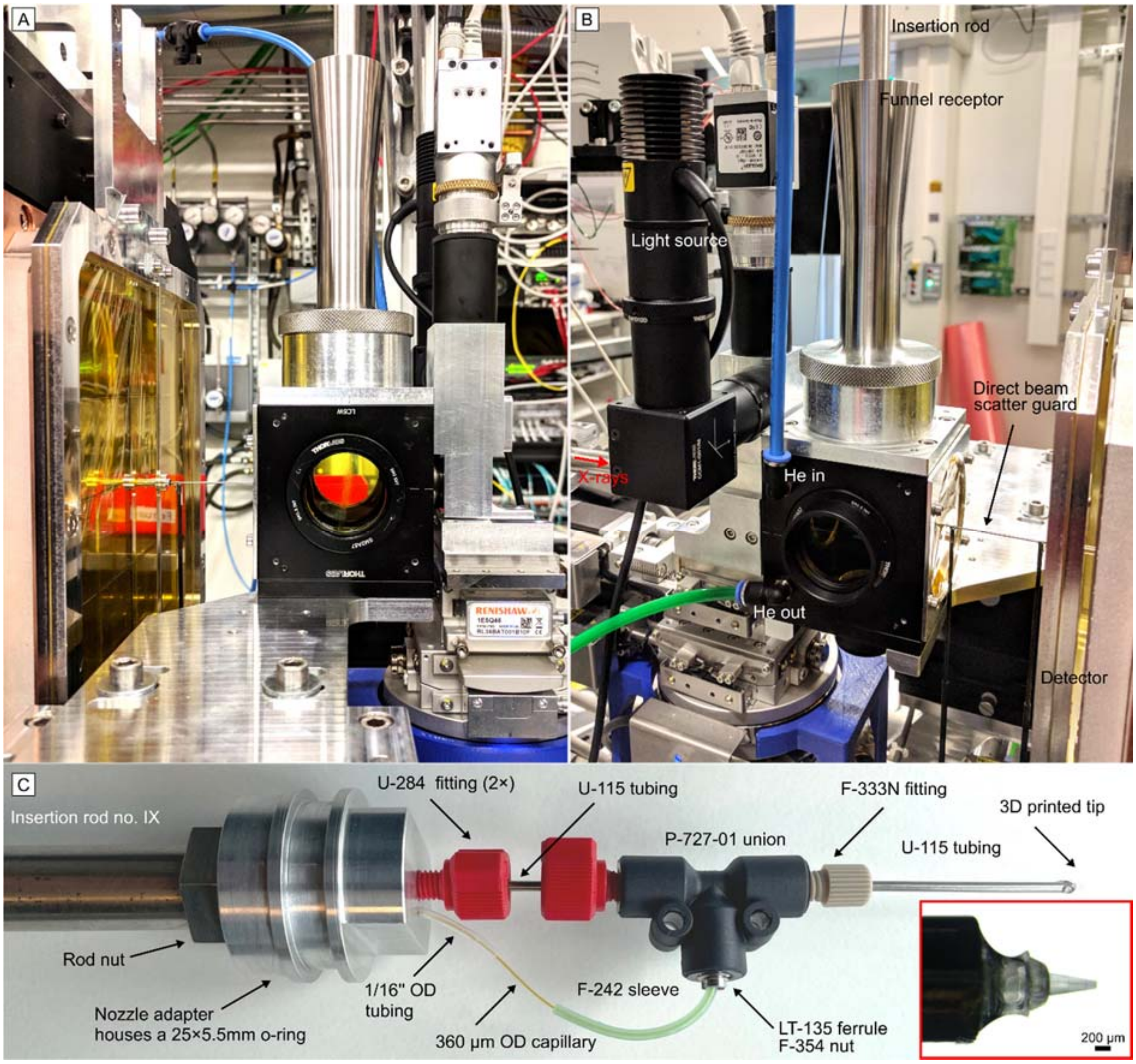

Figure S6 (A&B) Photographs of the SPB/SFX interaction region downstream (IRD) for high-viscous sample injection from two opposite perspectives. The use of wider ID capillaries (250 µm) allows the injector tip to be in distance (>10 cm) from a feeding sample syringe (not depicted here). The Kapton polyimide foil-sealed and helium-purged cube minimizes background signal during scattering. (C) Close-up of a 2PP–3D printed HVE injector assembly connected to a 30 cm long insertion rod (no. IX) with a description of utilized IDEX fluid connection parts. For a more uniform helium stream exiting only through the injector tip, the U-115 steel tubing in between the two red fittings can be exchanged with a F-242 sleeve. Mixing HVEs here can make use of dual-lumen sleeves (FEP MultiLumen, Zeus Industrial Products, Inc.) that encase two capillaries at once.

Table S3 Overview of nozzle/injection rods with details on their dimensions and connectivity. Injection hardware with GDVN-compatibility are catalogued with Arabic numerals, while HVE-rods are catalogued with Roman numerals.

**Rod no.**	**Rod dimensions**			**Bottom attachment**	**Connected adapter at bottom provides…**	**Receptor funnel**
	L [cm]	OD [in]	ID [in]			ID [mm]
1	120	0.5	0.25	Flange for 7.6×1.8mm O-ring	Fem. M22×1.5mm thread,[1)] ext. 25×4mm O-ring	32
2	120	0.5	0.25	Flange for 7.6×1.8mm O-ring	Fem. M22×1.5mm thread,[1)] ext. 25×4mm O-ring	32
3	120	0.5	0.25	Flange for 7.6×1.8mm O-ring	Fem. M22×1.5mm thread,[1)] ext. 25×4mm O-ring	32
4	120	0.5	0.25	Flange for 7.6×1.8mm O-ring	Fem. M22×1.5mm thread,[1)] ext. 25×4mm O-ring	32
5	120	0.5	0.25	Flange for 14×2mm O-ring	Fem. M22×1.5mm thread,[1)] ext. 25×4mm O-ring	32
6	120	0.5	0.25	Flange for 14×2mm O-ring	Fem. M22×1.5mm thread,[1)] ext. 25×4mm O-ring	32
7	120	0.5	0.37	Flange for 14×2mm O-ring	Fem. M22×1.5mm thread,[1)] ext. 25×4mm O-ring	32
8	120	0.5	0.37	Flange for 14×2mm O-ring	Fem. M22×1.5mm thread,[1)] ext. 25×4mm O-ring	32
9	120	0.5	0.37	Flange for 15×2mm O-ring	Fem. M22×1.5mm thread,[1)] ext. 25×4mm O-ring	32
10	120	0.5	0.25	Fem. M9×1mm thread, ext. 12.07×5.33mm O-ring	Male M9×1mm to fem. #10-32 UNF (nozzle holder)	22
11	120	0.5	0.25	Fem. M9×1mm thread, ext. 12.07×5.33mm O-ring	Male M9×1mm to fem. #10-32 UNF (nozzle holder)	22
12	120	0.5	0.25	Fem. M9×1mm thread, ext. 12.07×5.33mm O-ring	Male M9×1mm to fem. #10-32 UNF (nozzle holder)	22
13	120	0.5	0.25	Fem. M9×1mm thread, ext. 12.07×5.33mm O-ring	Male M9×1mm to fem. #10-32 UNF (nozzle holder)	22
14	120	0.5	0.25	Fem. M9×1mm thread, ext. 12.07×5.33mm O-ring	Male M9×1mm to fem. #10-32 UNF (nozzle holder)	22

I	103	0.5	0.25	Fem. M9×1mm thread	Male M9×1mm (Arizona or Heidelberg HVE injector), ext. 12.07×5.33mm O-ring	22
II	103	0.5	0.25	Fem. M9×1mm thread	Male M9×1mm (Arizona or Heidelberg HVE injector), ext. 12.07×5.33mm O-ring	22
III	103	0.5	0.25	Fem. M9×1mm thread	Male M9×1mm (Arizona or Heidelberg HVE injector), ext. 12.07×5.33mm O-ring	22
IV	103	0.5	0.25	Fem. M9×1mm thread	Male M9×1mm (Arizona or Heidelberg HVE injector), ext. 12.07×5.33mm O-ring	22
V	29.5	0.5	0.25	Fem. M9×1mm thread	Male M9×1mm (Arizona or Heidelberg HVE injector), ext. 12.07×5.33mm O-ring	22
VI	29.5	0.5	0.25	Fem. M9×1mm thread	Male M9×1mm (Arizona or Heidelberg HVE injector), ext. 12.07×5.33mm O-ring	22
VII	29.5	0.5	0.25	Fem. M9×1mm thread	Male M9×1mm (Arizona or Heidelberg HVE injector), ext. 12.07×5.33mm O-ring	22
VIII	29.5	0.5	0.25	Flange for 14×2mm O-ring	Fem. M22×1.5mm thread,[2)] ext. 25.5×5.5mm O-ring	35
IX	29.5	0.5	0.25	Flange for 14×2mm O-ring	Fem. M22×1.5mm thread,[2)] ext. 25.5×5.5mm O-ring	35
X	29.5	0.5	0.25	Flange for 14×2mm O-ring	Fem. M22×1.5mm thread,[2)] ext. 25.5×5.5mm O-ring	35

1) Compatible adapters further allow connection to the Cornell Mixing Injector Holder (see "Technical Drawings for Mixing Injector Holder Assembly and EuXFEL Nozzle Rod" within the supporting information for Calvey *et al.* 2019).

2) Compatible adapters allow further connection via fem. M9×1mm or fem. #10-32 UNF threads, respectively.

S4.4. Gas flow evaluation

The effect of high-pressure helium and liquid flows running through a GDVN (50–60–60 [µm]) was analysed at the IRU sample chamber at high vacuum conditions. The measurements (Fig. S7) were collected after pumping down the sample chamber for one hour, leading to starting values of 5.70×10^{-6} mbar (inside the chamber) and 1.30×10^{-6} mbar (inside the catcher shroud), respectively. The He flow rate has a much higher impact on the vacuum level inside the chamber, which must remain below 2×10^{-4} mbar to allow the operation of the detector. Furthermore, liquid jetting should be conducted in a way that the catcher pressure to chamber pressure ratio is kept ≤500. Fig. S8 depicts the catcher shroud in an opened sample chamber.

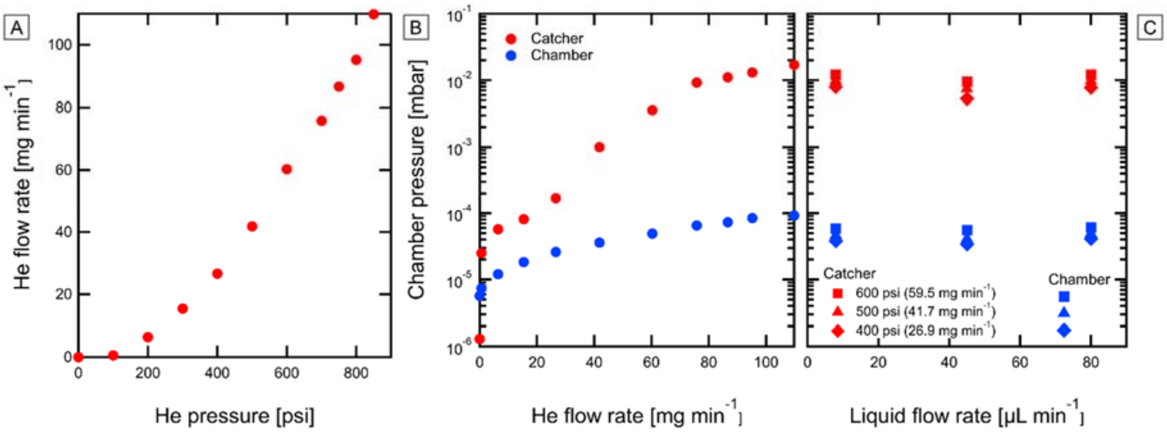

Figure S7 (A) Plot of the He flow rate as a function of the applied gas pressure when using a 50–60–60 [µm] GDVN, with capillary lengths of 2 m (100 µm ID). (B&C) Pressure measured inside of the evacuated catcher shroud (red) and outside (blue) of the catcher shroud within the sample chamber (B) during gas flow operation in absence of liquid flow and (C) with a combination of gas and liquid flow (*i.e.* conventional liquid jet operation) for three different gas/liquid flow rates. Reasonable gas flow rates for stable liquid jetting are 20–60 mg min^{-1}. No significant effect of the liquid flow on vacuum levels was observed.

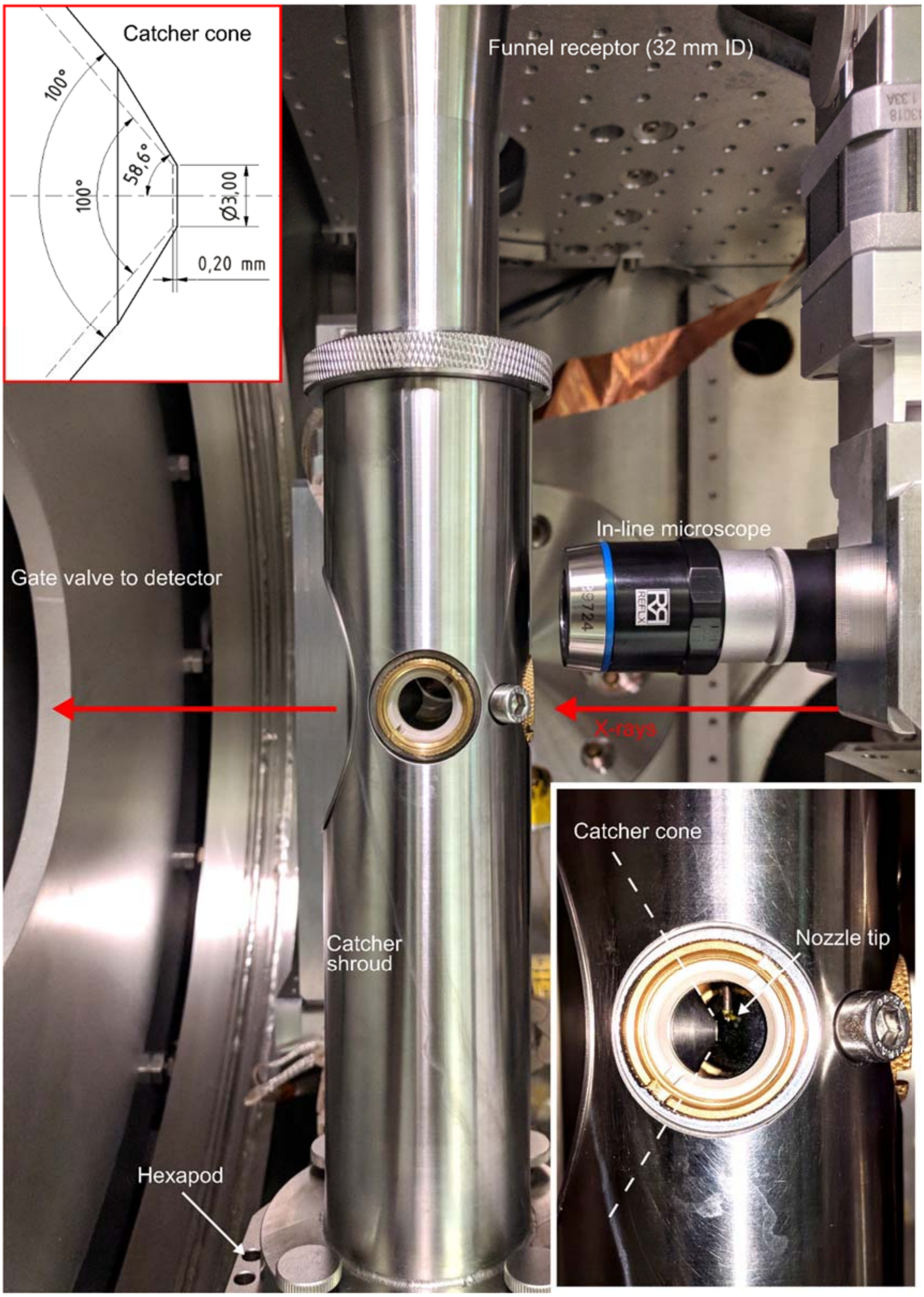

Figure S8 Photograph taken inside the SPB/SFX sample chamber at the IRU, showing the installed injector catcher system from the side view with details on the catcher re-entrant cone. An ideally cantered nozzle tip will initially be 1.056 mm away from the 100°-opened re-entrant cone which begins with a 3 mm wide through hole for the scattered photons.

S5. Lab-based jet characterization

Additional high-speed images were taken with a Photron Mini AX camera equipped with a 20× Plan Apo objective (Mitutoyo MY20X-804, 0.42 NA) and a LED (Schott KL 2500). While the jet diameter can be estimated, information on jet lengths and jet velocities cannot be evaluated due to motion blurring upon long exposure times. Fig. S9 shows an exemplary high-speed microscopy image.

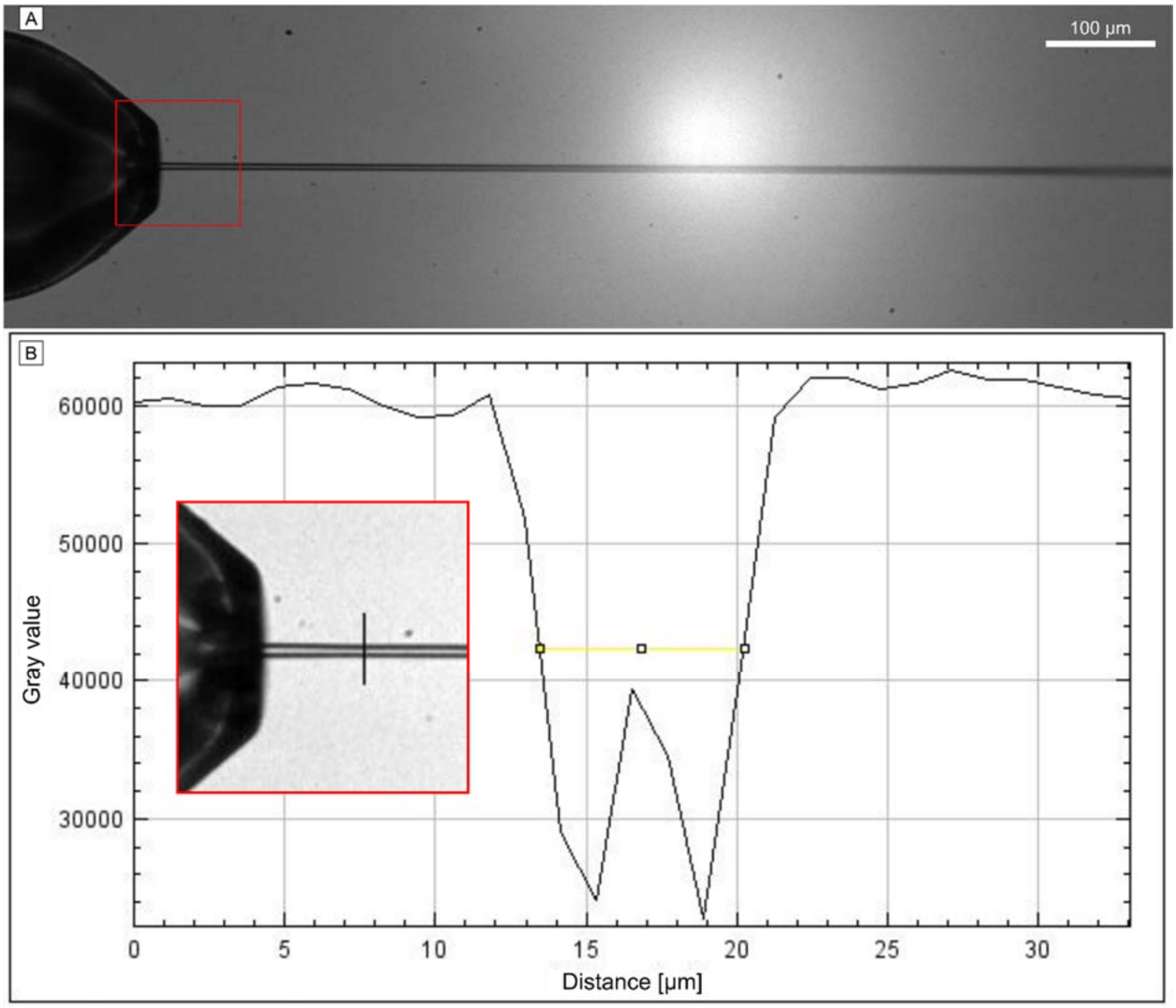

Figure S9 (A) High-speed camera image of a 30–30–30 [µm] GDVN that focuses water (here: Q = 70 µL min^{-1}) with pressurized He (300 psi, 13.8 mg min^{-1}) in a 1 mbar environment. The image of the liquid jet (20× magnification, 900×250 px^2 detection area, pixel size ~1.2 µm, 0.63 µm optical resolution) was taken with a frame rate of 10 000 s^{-1} and an exposure time of 5 µs. (B) Depiction of the manual determination of jet edges for a crude estimation of a 6.5 µm wide jet diameter (yellow line) within the program ImageJ. The beamline jet velocity experiment, however, revealed a more accurate diameter of 5.1 µm for this specific jet.

S6. Material properties

Today, a large number of photoresists for two-photon polymerization are available.(Schmid *et al.*, 2019; Huang *et al.*, 2020) The most prominent, IP-S (Nanoscribe), exhibits an enormous cross-linking density, hence allowing the creation of devices with unmatched mechanical, chemical, and thermal stability.(Bernardeschi *et al.*, 2021) Unfortunately, IP-S has a high autofluorescence in the visible range that prohibits fluorescence microscopy studies.(Knoška *et al.*, 2020) The IP-Visio variant does not have this disadvantage.(Ristok *et al.*, 2020) However, IP-Visio requires much shorter scan speeds and smaller slicing/hatching distances that significantly increase the overall printing times. Moreover, it is not optimized for small-orifice printing and showed only weak bonding to tubing materials assisted by conventional epoxy glues. Alternatively, OrmoComp (micro resist technology) can be used. This UV-sensitive mixture is composed of inorganic silicon alkoxide backbones with organic side groups connected by acrylic cross-linking agents,(Haas, 2000) and thus promises to combine the easy processability of polymers with desirable surface properties (e.g., high transparency, low sample adsorption).(Sikanen *et al.*, 2012) However, their inherent softness requires post-development curing steps prior to assembly. Another drawback is the material's apparent porosity: After microfluidic mixing experiments with Rhodamine 6G in hard-baked OrmoComp devices, residual fluorescent dye could be detected within the channel walls even after rigorous washing with water. Currently, we cannot exclude that these observations arise from adsorption effects. OrmoComps' advantage, however, lies in the high surface energy (originating from their glass-like nature) promoting good adhesion to metal tubings. Future developments may also lead to fully glass-based GDVNs out of a 2PP–3D printer.(Kotz *et al.*, 2021)

S6.1. Adhesion studies

As leak-free operation is indispensable, another crucial requirement the material has to fulfil, is strong and permanent adhesion to the feeding capillaries. To evaluate bonding strengths provided by various glues, 100 µm thick sheets of IP-S, IP-Visio and OrmoComp were fabricated: the liquid photoresists were UV-cured after dispersing them in between two PDMS blocks for 30 min (OrmoComp) and 60 min (IP-S, IP-Visio) using a UV LED (λ = 385 nm) curing chamber (XYZprinting, 3UD10XEU01K). The sheet thickness was governed by a set of double-layered Scotch strips serving as spacers between the PDMS blocks. Afterwards, the sheets were rinsed with isopropanol, blow dried and baked at 150 °C for 3 hours. The adhesion strengths between the cured sheets and prominent tubing materials (stainless steel, borosilicate glass, polyimide, each degreased with isopropanol) was investigated in combination with a variety of commercially available epoxy-based (Devcon 2-part epoxy, Loctite 3450) and UV-sensitive glues which are based on thiol-ene-networks (NOA81, NOA76 and NOA68 by Norland Products, Inc.). After glue attachment, the NOAs were cured for 30 min in the UV-curing chamber and then baked at 50 °C for 12 h. The epoxy glues were cured at RT

overnight (Loctite at 80 °C for 12 h). Bonding strengths (Table S4) were determined by how easily the structures could be disrupted manually. Furthermore, we present UV-Vis absorbance data of the same materials in Fig. S10.

Table S4 Overview of material properties and adhesive quality for different 2PP-photoresists and tubing materials suitable for the fabrication of microfluidic sample delivery device.

The adhesive quality is ranked qualitatively with ++ (very good), + (good), 0 (medium), - (poor), -- (very poor).

			Adhesion to					Material properties						
Part	Material name	Type	Devcon Epoxy	Loctite 3450	NOA81	NOA76	NOA68	Viscosity [Pa s]	Temp. stability [°C]	Price [€ per g]	Curing duration	Auto-fluorescence[2)]	Porosity/ adsorption effects[2)]	Trans-parency
Tubing	Borosilicate	Glass	++	++	+	0	+		~500			--	--	++
	Kapton	Polyimide	0	0	+	+	+		~200			--	--	0
	Stainless steel	Metal	++	++	+	0	0		~800			--	--	--
3D printed device[1)]	IP-S	Acrylate-based	-	+	++	-	++	13.6	286	6	+	++	--	+
	IP-Visio	Acrylate-based	-	-	-	-	-	unknown	282	13	0	--	--	++
	OrmoComp	Inorganic-org. hybrid	+	++	++	++	+	2	270	4	0	--	0	++
Adhesive	Devcon epoxy	Epoxy-based	++	++	+	-	+	10	93	0.3	-	+	+	+
	Loctite 3450	Epoxy-based	++	++	+	-	+	25	100	0.5	--	+	+	--
	NOA81	Thiol-ene-networks	+	+	++	++	++	0.3	125	1	++	--	--	+
	NOA76		-	-	++	++	++	4.5	90	3	+	--	+	++
	NOA68		-	-	++	++	++	5.0	90	1.2	++	--	+	++

1) Here as flat sheets

2) Here + means poor, while elsewhere it is a positive attribute

S6.2. UV-Vis absorption and fluorescence imaging

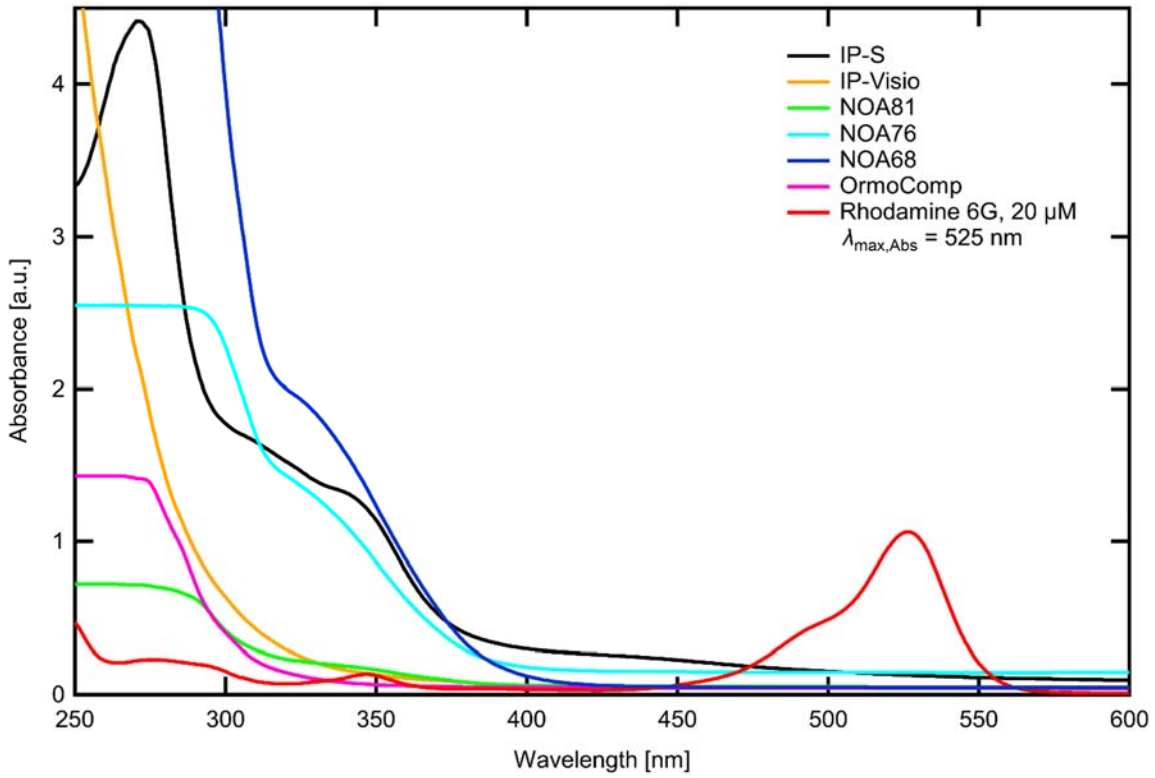

Figure S10 UV-Vis absorbance spectra for *ca.* 100 µm thick sheets of IP-S, IP-Visio, NOA81, NOA76, NOA68 and OrmoComp. The absorbance of a 20 µM Rhodamine 6G solution within a 10 mm thick cuvette is also shown. The optical absorption was measured with a Shimadzu UV-2700 spectrophotometer using a slit width of 2 nm, a signal accumulation time of 0.1 s and a sampling interval of 0.5 nm.

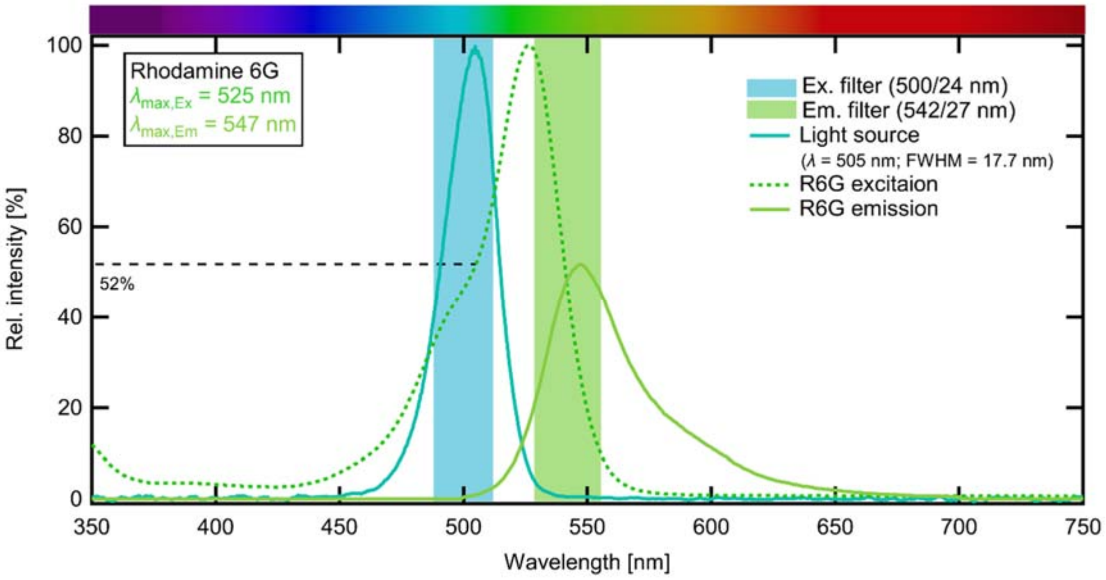

Figure S11 UV-Vis absorbance spectrum for the Rhodamine 6G dye with indicated spectral line of the light source (λ = 505 nm) and excitation/emission filter range. The optical absorption of Rhodamine 6G was measured as stated above in Fig. S10. The fluorescence emission spectrum of the dye was obtained from Thermo Fisher (Fluorescence SpectraViewer) and scaled to the experimental conditions.
